# Diabetic and hypertensive disorders following early pregnancy loss: a systematic review and meta-analysis

**DOI:** 10.1016/j.eclinm.2024.102560

**Published:** 2024-03-27

**Authors:** Jennifer Dunne, Damien Foo, Berihun A. Dachew, Bereket Duko, Amanuel T. Gebremedhin, Sylvester D. Nyadanu, Gavin Pereira, Gizachew A. Tessema

**Affiliations:** a Curtin School of Population Health, Faculty of Health Sciences, Curtin University, Kent Street, Bentley, Western Australia, 6102, Australia; b enAble Institute, Curtin University, Kent Street, Bentley, Western Australia, 6102, Australia; c Yale School of the Environment, Yale University, New Haven, CT, United States; d Australian Centre for Precision Health, UniSA Clinical and Health Sciences, University of South Australia, North Terrace, Adelaide, South Australia, 5000, Australia; e School of Nursing and Midwifery, Edith Cowan University, Joondalup Campus, Joondalup, Western Australia, 6027, Australia; f School of Public Health, University of Adelaide, Adelaide, South Australia, 5000, Australia

**Keywords:** Miscarriage, Abortion, Diabetes, Hypertension, Pre-eclampsia

## Abstract

**Background:**

Spontaneous and induced abortions are common outcomes of pregnancy. There is inconsistent evidence of an association between early pregnancy loss and subsequent diabetic and hypertensive disorders in women. This systematic review and meta-analysis evaluated evidence on the risk of the subsequent development of pregnancy and non-pregnancy related diabetic and hypertensive disorders in women who experienced an early pregnancy loss.

**Methods:**

Systematic searches were conducted in seven electronic databases (CINAHL Plus, Ovid/EMBASE, Ovid/MEDLINE, ProQuest, PubMed, Scopus, and Web of Science) from inception to 22nd December 2023. Studies were included if they reported an exposure of spontaneous abortion (SAB), induced abortion (IA) or recurrent pregnancy loss (RPL) with an outcome of gestational diabetes mellitus, pre-eclampsia, gestational hypertension, and non-pregnancy related diabetic and hypertensive disorders. Risk of bias was assessed using Risk of Bias Instrument for Non-Randomized Studies of Exposures (ROBINS-E). Random effects meta-analysis was used to pool odds of developing diabetic and hypertensive disorders following an early pregnancy loss. This study is registered with PROSPERO (CRD42022327689).

**Findings:**

Of 20,176 records, 60 unique articles were identified for full-text review and 52 met the inclusion criteria, representing a total population of 4,132,895 women from 22 countries. Thirty-five studies were suitable for meta-analysis, resulting in a pooled odds ratio (OR) of 1.44 (95% confidence interval (CI) 1.23–1.68) for gestational diabetes mellitus following a prior SAB and a pooled OR of 1.06 (95% CI 0.90–1.26) for pre-eclampsia following a prior SAB. RPL increased the odds of developing pre-eclampsia (OR 1.37 95% CI 1.05–1.79). There was no association between IA and diabetic and hypertensive disorders.

**Interpretation:**

A prior SAB was associated with increased odds of gestational diabetes mellitus, but not pre-eclampsia. However, women who experienced RPL had an increased risk of subsequent pre-eclampsia. Future research is required to establish evidence for an association between early pregnancy loss with non-pregnancy related diabetic and hypertensive disorders.

**Funding:**

10.13039/501100000925National Health and Medical Research Council.

**Research in context**

**Evidence before this study**

Early pregnancy loss is a common occurrence during pregnancy, which can have short- and long-term health consequences in the subsequent pregnancy and beyond. We undertook a comprehensive search of the literature from inception to 22nd December 2023 for studies related to early pregnancy loss and subsequent diabetic and hypertensive disorders. Recent systematic reviews and meta-analyses have limited to providing an aggregate summary of the association between spontaneous abortion (SABs) and induced abortion (IA) with the subsequent development of gestational diabetes mellitus, indicating a lack of comprehensive systematic review that synthesised available data across all pregnancy and non-pregnancy related diabetic and hypertensive disorders.

**Added value of this study**

This was a comprehensive assessment of the association between SAB, IA, and recurrent pregnancy loss (RPL) with the subsequent development of pregnancy (gestational diabetes; pre-eclampsia; gestational hypertension) and non-pregnancy diabetic (type 2 diabetes) and hypertensive disorders. Our findings support the association between SAB and the development of gestational diabetes mellitus and gestational hypertension in a subsequent pregnancy. Of significance, was the women who experienced a prior RPL had an association with subsequent pre-eclampsia; yet this association was not evident for women who had one prior SAB.

**Implications of all the available evidence**

The combined evidence indicates a need for further research to understand the biological mechanisms linking spontaneous abortion with pre-eclampsia, particularly for women who experience RPLs. More research is also required to investigate associations between early pregnancy loss events and the subsequent development of non-pregnancy related diabetic and hypertensive disorders.

## Introduction

Early pregnancy losses are not an uncommon outcome of pregnancy, with spontaneous and induced abortions frequent outcomes. Spontaneous abortion (SAB), also known as miscarriage, occurs in approximately 15–25% of all pregnancies, with an estimated 23 million SABs occurring globally each year.^1^ SAB generally refers to a loss of a pregnancy without intervention prior to a pre-specified gestational period, which varies from high-income countries (<20–22 weeks of gestation or birthweight <400–500 g)^2^ to low and middle-income countries (<28 weeks of gestation or birthweight <1000 g).^3^ It is estimated that 1–2% of women experience recurrent pregnancy loss (RPL), defined as two or more consecutive pregnancy losses (SABs).^4^ Induced abortions (IAs) are the most common medical intervention to terminate a pregnancy, with the annual global rates estimated to be as high as 121 million.^5^ Most IAs are reported to result from unintended pregnancies.^5^

It has been established that pregnancy complications are a strong risk factor for the future development of cardiovascular disease.^6^^,^^7^ Women with a history of pregnancy related hypertensive disorders (i.e., pre-eclampsia/eclampsia and gestational hypertension)^8^^,^^9^ and gestational diabetes mellitus^10^ have a greater overall risk of cardiovascular disease compared to women who experience a healthy pregnancy. Furthermore, women who have experienced a SAB are at greater risk for the subsequent complications of pregnancy, such as placental abruption, fetal growth restriction, preterm birth and stillbirth,^11^ all of which are associated with the subsequent increased risk of cardiovascular disease. More recently, there has been an increased interest in the potential impacts of early pregnancy loss on the subsequent development of diabetic^12^, ^13^, ^14^, ^15^, ^16^, ^17^, ^18^ and hypertensive disorders^14^^,^^18^ in women, with both disorders considered putative risk factors to the development of cardiovascular disease.^19^ The biological mechanism for the increased cardiovascular and metabolic risk is unclear; however, it could be due to oxidative stress and inflammation^20^ which could also contribute to other early pregnancy loss^21^ and other health outcomes, such as gestational diabetes mellitus. An alternative hypothesis may be that exposure to an early pregnancy loss could initiate an immunological cascade that could lead to the subsequent development of diabetic and hypertensive disorders, such as type-2 diabetes.^15^ However, there remains limited evidence to support the association between early pregnancy loss and the subsequent risk of developing a diabetic or hypertensive disorder. Of this limited evidence, one systematic review and meta-analysis^12^ examined the association between a prior SAB or stillbirth and the risk of developing diabetes. Their findings indicated a 62% increased risk of gestational diabetes mellitus and a 15% risk of non-pregnancy diabetes compared to women who never experienced a SAB; however, IAs were not included. A more recent meta-analyses found that a history of either SAB (OR 1.67 95% confidence interval (CI) 1.30–1.78) or IA (OR 1.07 95% CI 1.03–1.11) increased the risk of gestational diabetes mellitus in a subsequent pregnancy; however, 29 of the 31 included studies were from low- and middle-income countries suggesting potential homogeneity influencing their findings. Currently, the evidence for the association between early pregnancy loss and subsequent hypertension has not been systematically examined.

Based on the hypothesis that an early pregnancy loss may increase the risk of subsequent diabetic or hypertensive disorders, the aim of this systematic review was to comprehensively review the current observational evidence for the effect of early pregnancy loss (SABs, IAs, RPLs), on pregnancy and non-pregnancy-related diabetic and hypertensive disorders in women. The evidence obtained from this review is important to inform families and clinicians regarding reproductive, cardiovascular, and metabolic health, particularly in relation to women who have experienced an early pregnancy loss.

## Methods

### Search strategy and selection criteria

We followed the recommendations by the Preferred Reporting for Systematic Review and Meta-Analysis (PRISMA) 2020^22^ and the Meta-analyses of Observational Studies in Epidemiology (MOOSE)^23^ reporting guidelines to conduct this systematic review and meta-analysis. The literature search strategy, identification of relevant studies, data extraction and analysis were compiled in accordance with a predefined published protocol,^24^ and a prospective protocol registration from the International Prospective Register of Systematic Reviews (PROSPERO) (Registration # CRD42022327689). We searched peer-reviewed literature for articles investigating the association between early pregnancy loss and the subsequent development of pregnancy and non-pregnancy related diabetic and hypertensive disorders using CINAHL Plus, Ovid/EMBASE, Ovid/MEDLINE, ProQuest, PubMed, Scopus, and Web of Science databases. The initial databases were searched on April 15th 2022, with an updated search conducted on the 22nd December 2023 using the same search criteria. Search strategies for each database used medical subject headings (MeSH) terms and keywords related to the exposure and outcomes (Supplementary Table S1). We also undertook a search on Google Scholar to identify grey literature. We further searched the reference lists of in the included studies for relevant records not previously captured by the electronic search.

Studies were eligible for inclusion if the: (1) population includes women of reproductive age or post-menopausal women with no prior diabetic or hypertensive co-morbidities before the current pregnancy; (2) study design includes all observational studies such as cross-sectional, case–control and cohort studies; (3) exposure criterion includes studies that investigated history of early pregnancy loss (SAB, IA, RPL) as the primary exposure; and, (4) outcome criterion includes studies that investigated at least one diabetic (gestational diabetes mellitus, type 1 diabetes or type 2 diabetes) or hypertensive (pre-eclampsia, gestational hypertension and non-pregnancy hypertension) outcome. We excluded studies based on the following three criteria: (1) non-primary studies including case reports and series, commentaries, editorials, letters to the editors or reviews; (2) studies published in languages other than English; (3) studies with incomplete information on effect estimates (e.g., missing confidence intervals and did not report sufficient data to calculate the effect estimates).

### Procedures

Two reviewers (DF, JD) used EndNote X10 to screen and review the titles and abstracts of all the identified records retrieved during the searches. Four reviewers (DF, JD, SDN, BAD) performed the full-text review of the identified articles and extracted the data. The reason for exclusion at full text review was documented (Supplementary Table S2). When conflicts for including or excluding articles between the two reviewers occurred, an independent reviewer (GAT) was involved for a final decision. Where critical data was missing, the relevant corresponding authors were contacted. If multiple studies published using the same cohort, the reporting outcomes from the most recent study were included. For each included study, data was independently extracted by at least two reviewers (DF, JD, SDN, BAD) using a *priori* developed data extraction format which included: first author and year published, geographical setting, study design, sample size, population demographics, definitions and ascertainment of exposure and outcomes, effect estimates with its confidence intervals, and adjustment variables included in the model.

### Statistics

Risk of bias was independently assessed for the included studies by at least two reviewers (JD, BA) using the Risk of Bias in Non-randomized Studies of Exposure (ROBINS-E) tool.^25^ The tool has seven domains that assess sources of potential bias: confounding, selection of participants, classification of exposures, deviation from intended exposures, missing data, measurement of outcomes, and selection of the report result. Domains were assessed as low risk, some concerns, high risk or very high risk of bias; with each study rated overall to the same level of severity of the highest risk of the risk assessed in an individual domain.^26^ Where there were two or more domains that reported a high risk of bias, they were allocated an overall very high risk of bias.^26^ We rated a study as having a high risk of bias if there was no control for confounding. When there were differences in the risk of bias assessment, an independent reviewer (GAT) made a final decision. The use of ROBINS-E tool (released 20th June 2023) to assess the risk of bias in observational studies was a deviation from our published protocol in which we had anticipated using GRADE, a critique of which was the downgrading of non-randomised studies by assigning them a starting rating of ‘low certainty, confidence or quality’.^26^

We performed a narrative and textual synthesis of all included studies, with all extracted data of interest provided in tabular format. Based on the exposure status (SAB, IA and RPL), study-level effects were estimated on outcomes of gestational diabetes mellitus, pre-eclampsia, gestational hypertension, hypertensive disorders of pregnancy, and non-pregnancy diabetes or non-pregnancy hypertensive disorders. Raw frequency data was used to estimate the odds of early pregnancy loss on diabetic or hypertensive disorders. We estimated the pooled adjusted effects comparing the OR of early pregnancy loss with the outcomes using a random effect sub-group meta-analysis with the inverse variance method. We applied random effects estimate meta-analysis with DerSimonian and Laird estimator, as this allowed each study to estimate different effect size and allowed for a more balanced approach to weighting in the estimation of the pooled effect. To quantify the statistical heterogeneity between studies, we reported the *I*^2^ statistic, estimated as *I*^2^ = 100% (Q–df)/Q, where Q is Cochrane's heterogeneity statistic and df represents the degrees of freedom.^27^ *I*^2^ indicates the percentage of total variation across studies due to true variation rather than chance, providing a better powered estimate of heterogeneity in cases where few studies are included. Interpretation of the *I*^2^ statistic was made consistent with international guidelines.^28^ Publication bias was assessed by funnel plot and Egger's test to evaluate the small study effects. To explore possible sources of heterogeneity, we employed a random-effect meta-regression model considering year of publication for exposure-outcome associations in which there were eight or more included studies. Analysis were performed in R4.3.0.

### Ethics

Due to the nature of the present study, informed consent or approval by local ethical committees was not required.

### Role of funding source

The funders of the study had no role in the study design, data collection, data analysis, data interpretation, or writing of the study.

## Results

**Fig. 1:**
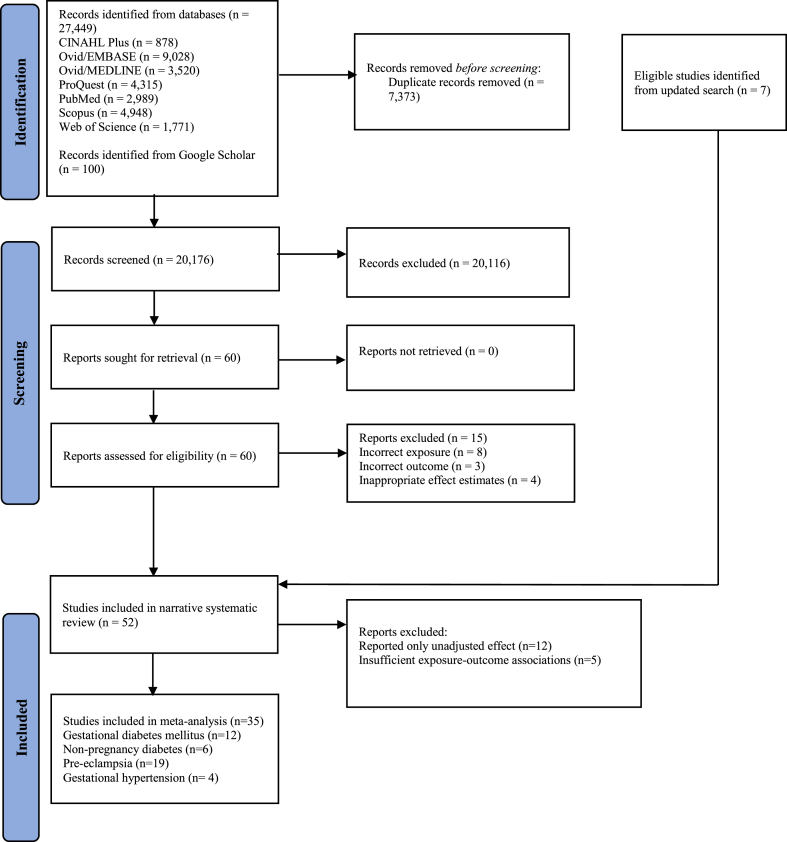
Systematic review of the literature examining the association between early pregnancy loss and pregnancy and non-pregnancy diabetic and hypertensive disorders.

In total, 27,549 records were retrieved from databases and Google Scholar. After removing duplicates and subsequent screening for title, abstract and full text, 60 studies were deemed potentially eligible (Fig. 1). Upon full-screening, 15 articles were excluded, and a further seven studies were identified following an updated review, leaving a total of 52 articles for the final review. The most common reasons for exclusion were due to the incorrect or unclear definition of the outcome (*n* = 8), followed by four studies that did not report appropriate effect estimates and three studies that did not have a clear outcome of interest. The detailed flowchart of the literature search process is shown in Fig. 1.

### Characteristics of the included studies

The 52 included studies contained 4,132,895 women from 22 countries. Sixteen of the studies were from Asia of which 12 studies were from China, 14 were from Europe, ten from North America, seven from the Middle East, and five studies were from Africa. Of the study designs, 64% (n = 33) were cohort studies and a quarter (n = 13) were case–control designs. Twenty-four studies examined the association between early pregnancy loss and diabetic disorders, thirty-seven studies examined the association between early pregnancy loss and hypertensive disorders, and nine studies examined the association between early pregnancy loss and both hypertension and diabetic disorders (Supplementary Table S1).

#### Risk of bias

**Fig. 2:**
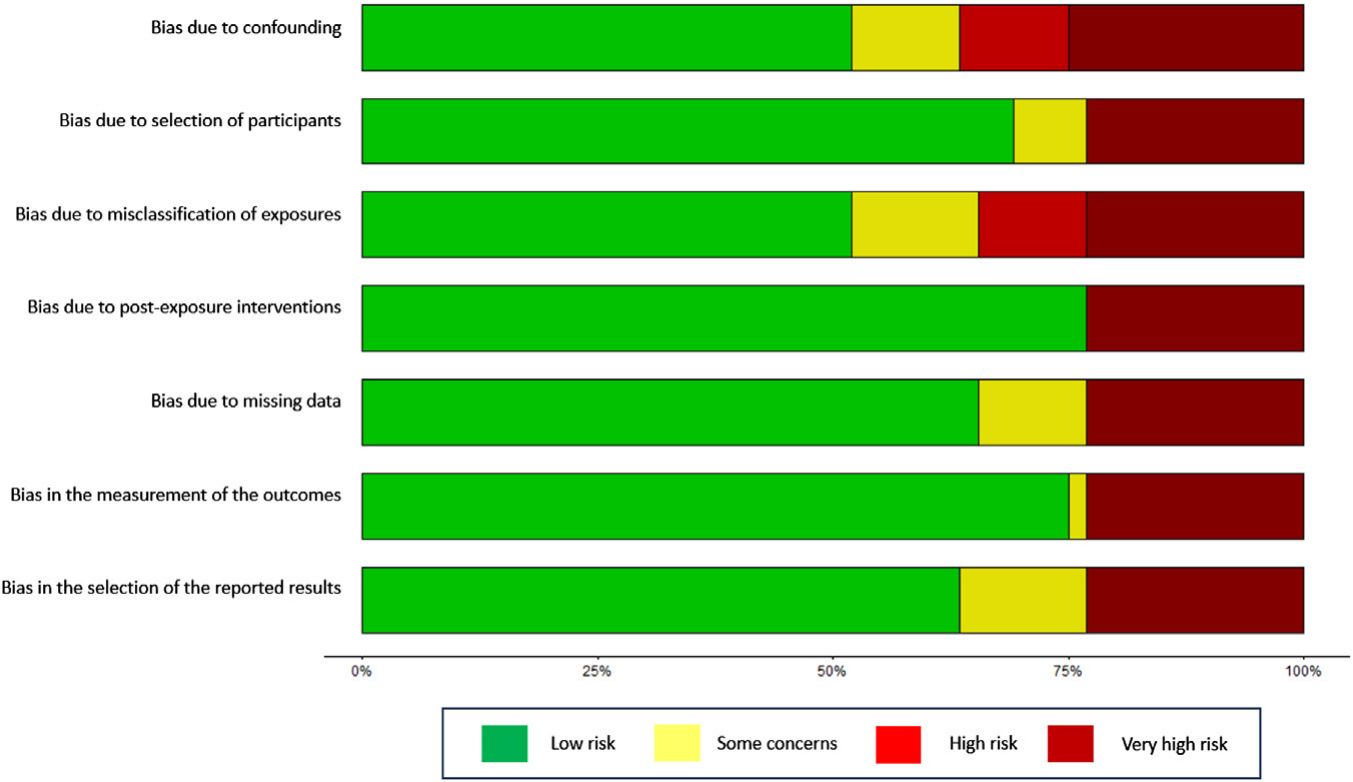
Risk of Bias in Non-randomized Studies of Exposure (ROBINS-E) summary. OR: Odds ratio. CI: Confidence interval, p: p-value.

Critical risk of bias was found for confounding with 12 studies,^29^, ^30^, ^31^, ^32^, ^33^, ^34^, ^35^, ^36^, ^37^, ^38^, ^39^, ^40^ that did not adjust for confounding given an overall score assessment of very high risk using ROBINS-E. An additional study^15^ scored very high risk due to lack of confounding variables. Ten studies were marked as high risk of bias, six^41^, ^42^, ^43^, ^44^, ^45^, ^46^ of which failed to control for adequate confounding variables and four^47^, ^48^, ^49^, ^50^ which failed to sufficiently define their exposure variables.^41^, ^42^, ^43^, ^44^, ^45^, ^46^, ^47^, ^48^, ^49^, ^50^ Thirteen studies^14^^,^^17^^,^^51^, ^52^, ^53^, ^54^, ^55^, ^56^, ^57^, ^58^, ^59^, ^60^, ^61^ were deemed to be of moderate risk of bias, with 16 studies deemed to be of low risk of bias^16^^,^^18^^,^^33^^,^^62^, ^63^, ^64^, ^65^, ^66^, ^67^, ^68^, ^69^, ^70^, ^71^, ^72^, ^73^, ^74^ (Fig. 2).

Within the included studies, exposure definitions were slightly varied, with studies that primarily investigated risk factors for diabetic or hypertensive disorders less likely to define their exposures (e.g., eight studies stated abortion as the exposure without specification for gestational age cutoffs). IA exposure was investigated in 16 included studies and in nine studies RPL was the exposure. One study^50^ mixed SAB and IA together under abortion, while another study^40^ report an exposure of recurrent abortion in which abortion was undefined. With the exception of the 18 studies that had access to previously recorded medical data, ascertainment of the exposure variable was derived from self-report either through an interview or questionnaire. The outcomes for the diabetic (type 1 diabetes, type 2 diabetes and gestational diabetes mellitus) and hypertensive disorders (gestational hypertension, pre-eclampsia/eclampsia, and non-pregnancy hypertension) were diagnosed clinically by medical experts (see Supplementary Table S5).

#### Association between early pregnancy loss and gestational diabetes mellitus

**Fig. 3:**
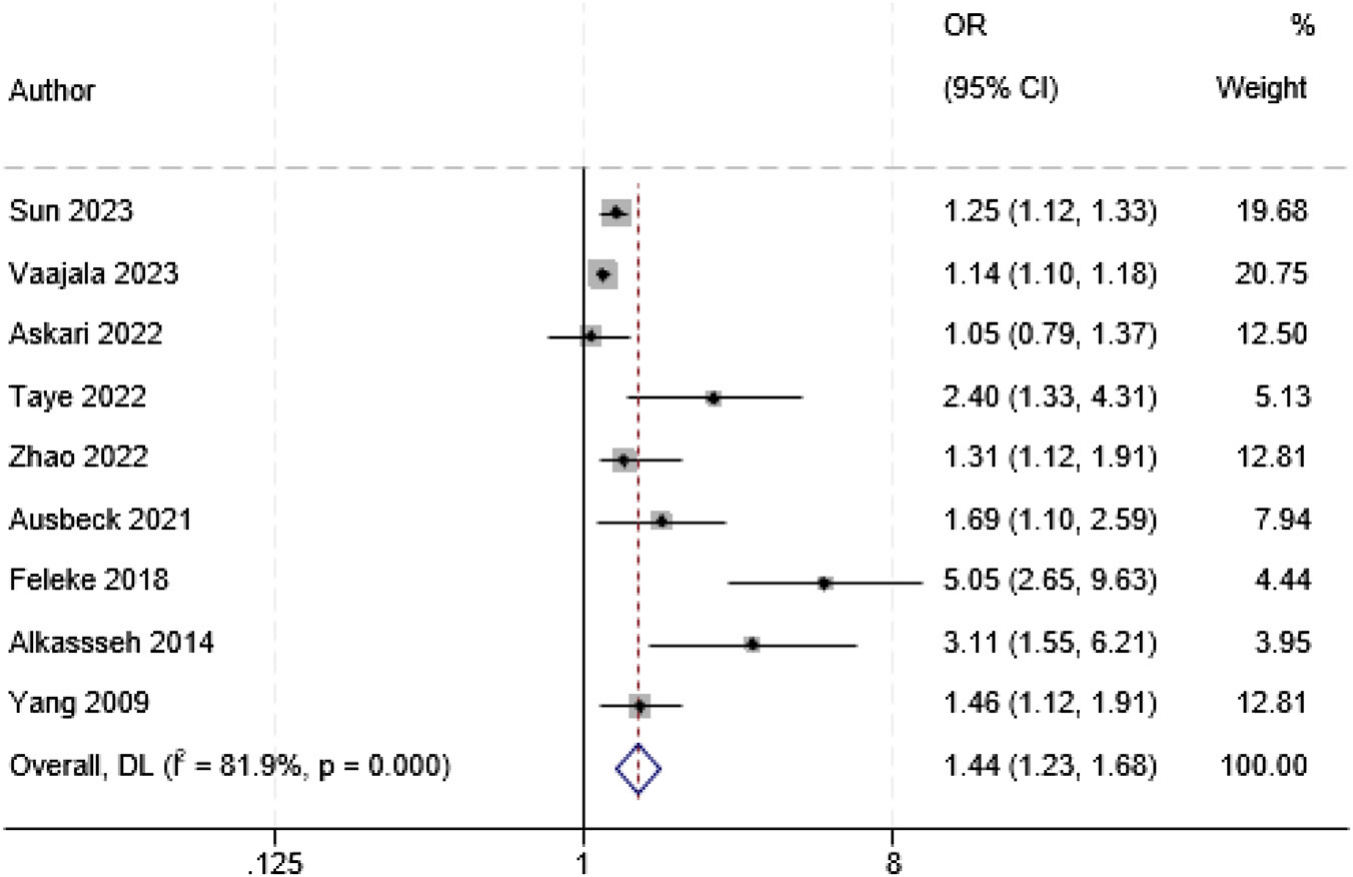
Meta-analysis of the association between a prior spontaneous abortion and the risk of developing gestational diabetes mellitus in a subsequent pregnancy. OR: Odds ratio. CI: Confidence interval, p: p-value.

Among 12 studies that provided effect estimates for the risk of subsequent gestational diabetes mellitus following a previous early pregnancy loss, most reported an increase in effect estimate from weak to strong, with adjusted odds ratio (aOR) ranged from 1.05 (95% 0.80–1.37) to a 5.05 (95% 2.65–9.63) for women who had experienced a prior SAB. Nine studies^17^^,^^44^^,^^47^, ^48^, ^49^^,^^59^^,^^60^^,^^62^^,^^73^ that examined the association between SAB and gestational diabetes mellitus reported adjusted effect estimates and were deemed eligible for inclusion in the meta-analysis. The pooled estimates of these studies showed that women who experienced a previous SAB had 44% increased odds of developing gestational diabetes mellitus in a subsequent pregnancy (pooled OR 1.44; 95% CI 1.23–1.68) (Fig. 3). While there was substantial heterogeneity between studies (*I*^*2*^ = 81.9%), the funnel plots (Supplementary Figure S10) and Egger's test (p-value = 0.11) indicated that there was no evidence of publication bias. The random-effect meta-regression model found that publication year did not have a detectable significant linkage with heterogeneity (Coefficient = 0.98, p-value = 0.42).

For women who had a prior IA,^17^^,^^30^^,^^33^^,^^59^^,^^60^ there was a small increased risk of gestational diabetes mellitus in subsequent pregnancies, with effects ranging from aOR 1.03 (95% CI 0.97–1.10)^59^ to aOR 1.18 (95% CI 1.12–1.24).^17^ Three studies^17^^,^^59^^,^^60^ were included in a meta-analysis, producing a pooled aOR of 1.04 (95% CI 0.97–1.12, *I*^*2*^ = 89.6%), indicating that women who had a prior IA did not have increased odds of developing gestational diabetes mellitus in subsequent pregnancy (Supplementary Figure S1). Of the six studies^41^^,^^42^^,^^45^^,^^48^^,^^62^^,^^75^ that examined the association between RPL and gestational diabetes mellitus effects ranged from an aOR of 0.69 (95% CI 0.47–1.02) to aOR 1.69 (95% CI 1.10–2.59).^62^ Five studies were included in the meta-analysis producing an aOR of 1.09 (95% CI 0.89–1.32, *I*^*2*^ = 70.5%), suggesting that women who experienced RPL do not have increased odds of developing gestational diabetes mellitus in subsequent pregnancy (Supplementary Figure S2).

#### Association between early pregnancy loss and non-pregnancy diabetes

Seven studies^14^, ^15^, ^16^^,^^18^^,^^53^^,^^56^^,^^76^ reported conflicting associations between SAB and the subsequent development of a non-pregnancy diabetic disorder. One study^14^ reported an adjusted incidence rate ratio (IRR) of 1.25 (95% CI 1.15–1.36) for the association between SAB and either type 1 or type 2 diabetes. Four studies^15^^,^^18^^,^^53^^,^^56^ examined the association between SAB and the development of type 2 diabetes, ranging from aOR of 1.02 (95% CI 0.94–1.10) to aOR 1.40 (95% CI 1.25–1.58). Five studies^15^^,^^16^^,^^53^^,^^56^^,^^76^ were included in the meta-analysis with a pooled aOR of 1.09 (95% CI 0.97–1.24; *I*^*2*^ = 83.3%) (Supplementary Figure S3). Two studies^15^^,^^18^^,^^53^^,^^56^ reported the association between IA and type 2 diabetes (aOR 0.97, 95% CI 0.87–0.02)^18^ to aOR 1.06, 95% CI 1.02–1.11), while one study^16^ reported the association between IA and either type 1 or type 2 diabetes (aOR 0.71, 95% CI 0.56–0.90). The pooled effect for the association between IA and the development of a non-pregnancy diabetes was aOR 0.94 (95% CI 0.80–1.11; *I*^*2*^ = 84.6%) (Supplementary Figure S4). No studies reported an association between RPL and the development of non-pregnancy related diabetes.

#### Association between early pregnancy loss and pre-eclampsia

**Fig. 4:**
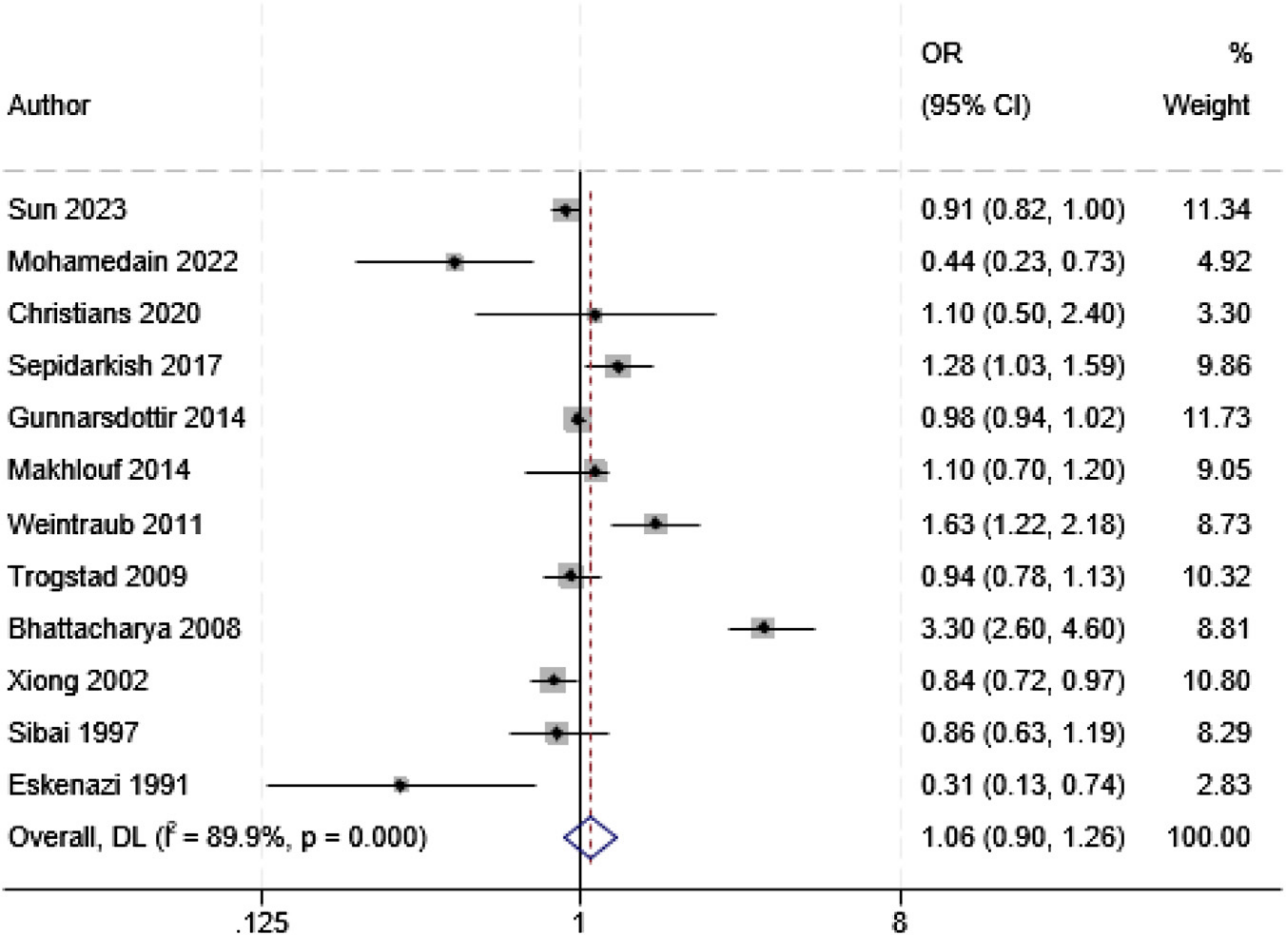
Meta-analysis of the association between a prior spontaneous abortion and the risk of developing pre-eclampsia in a subsequent pregnancy.

Among the studies that examined the risk of pre-eclampsia in a subsequent pregnancy following a prior SAB (n = 16),^29^^,^^32^^,^^46^^,^^51^^,^^52^^,^^54^^,^^57^, ^58^, ^59^^,^^63^, ^64^, ^65^^,^^68^, ^69^, ^70^^,^^72^ there was conflicting evidence of risk, with effect sizes ranged from a protective aOR of 0.31 (95% CI 0.13–0.74) to an elevated risk (aOR 3.30; 95% CI: 2.60–4.60). Twelve studies^46^^,^^51^^,^^52^^,^^54^^,^^57^, ^58^, ^59^^,^^63^, ^64^, ^65^^,^^70^^,^^72^ were eligible for inclusion in the meta-analysis producing a pooled effect of aOR 1.06 (95% CI 0.90–1.26; *I*^*2*^ = 89.9%) (Fig. 4). The funnel plots (Supplementary Figure S11) and Egger's test (p-value = 0.43) indicated that there was no evidence of publication bias. The random-effect meta-regression model found that publication year did not have a detectable significant linkage with heterogeneity (Coefficient = 1.01, p-value = 0.26).

The association between a prior IA and subsequent pre-eclampsia was also inconclusive, with seven studies^32^^,^^34^^,^^37^^,^^52^^,^^59^^,^^69^^,^^71^ reporting estimates ranging from aOR of 0.61 (0.38–0.97) to aOR 10.8 (1.20–29.10).^52^ The four studies^52^^,^^59^^,^^69^^,^^71^ included in the meta-analysis produced a pooled aOR of 0.91 (95% CI 0.68–1.22; *I*^*2*^ = 76.69%) (Supplementary Figure S5). Of the studies^31^^,^^36^^,^^41^^,^^42^^,^^45^^,^^62^^,^^67^^,^^75^ that examined an association between RPL and the subsequent odds of developing pre-eclampsia (n = 8), effects ranged from aOR 0.87 (95% CI 0.56–1.36)^62^ to aOR 2.81 (95% CI 0.95–8.27).^41^ Six studies^41^^,^^42^^,^^45^^,^^62^^,^^67^^,^^75^ produced a pooled effect of aOR 1.37 (95% CI 1.05–1.79; *I*^*2*^ = 70.1%) (Supplementary Figure S6), indicating that women who experience a RPL have increased odds of developing pre-eclampsia in their subsequent pregnancy.

#### Association between early pregnancy loss and hypertensive disorders of pregnancy

Twelve studies^31^^,^^32^^,^^36^^,^^40^^,^^42^^,^^46^^,^^50^^,^^55^^,^^59^^,^^66^^,^^68^^,^^69^ examined an association between early pregnancy loss and the subsequent odds of developing gestational hypertension, three^50^^,^^59^^,^^69^ of which also reported an association with hypertensive disorders of pregnancy. When the exposure was SAB, studies^32^^,^^46^^,^^50^^,^^55^^,^^59^^,^^66^^,^^68^ that reported the odds of subsequently developing gestational hypertension ranged from aOR 1.00 (95% CI 0.6–1.7)^55^ to aOR 1.41 (95% CI 1.07–1.85).^46^ The three studies included in the meta-analysis produced an effect of aOR 1.18 (95% CI 1.04–1.33; *I*^*2*^ = 0%) (Supplementary Figure S7), indicating that women who experience a prior SAB had increased odds of developing gestational hypertension in a subsequent pregnancy. Only one study^59^ reported an association between SAB and a hypertensive disorder of pregnancy, with an aOR of 1.02 (95% CI 0.93–1.12).

Four studies^32^^,^^55^^,^^59^^,^^69^ examined an association between IA and the subsequent development of gestational hypertension with effects ranging from aOR 0.73 (95% CI 0.49–1.10) to aOR 1.19 (95% CI 1.02–1.39), producing a pooled effect of aOR 0.94 (95% CI 0.80–1.10; *I*^*2*^ = 5.4%) (Supplementary Figure S8). Of the three studies^31^^,^^36^^,^^42^ that reported an association between RPL and the subsequent risk of developing gestational hypertension, only one^42^ reported adjusted effect size of aOR 1.14 (95% CI 0.98–1.28). Evidence of the association between IA with the subsequent development of a hypertensive disorder of pregnancy was limited to one^59^ study, with aOR of 0.96 (95% CI 0.87–1.06). No studies reported an adjusted effect of RPL and a hypertensive disorder of pregnancy.

#### Association between early pregnancy loss and hypertensive disorders

Four studies^14^^,^^18^^,^^43^^,^^61^ examined the association between an early pregnancy loss and the odds of developing non-pregnancy hypertension. In general, a marginal increased risk was observed, with three studies^14^^,^^18^^,^^43^ reporting adjusted effects for the association between SAB and non-pregnancy hypertension, ranging from aIRR 1.07 (95% CI 1.02–1.12)^14^ to aIRR 1.15 (95% CI 0.99–1.34).^43^ There was insufficient evidence to undertake a meta-analysis. Two studies^18^^,^^73^ reported conflicting evidence for the association between IA and non-pregnancy, with adjusted aOR ranging from a protective effect aOR 0.88 (95% CI 0.84–0.92)^18^ to a risk of aOR 1.19 (95% CI 1.02–1.39),^61^ producing a pooled effect of aOR 1.09 (95% CI 0.75–1.36; *I*^*2*^ = 92.6%) (Supplementary Figure S9). No studies reported an adjusted effect of RPL and non-pregnancy hypertension.

## Discussion

Evidence from this systematic review and meta-analysis found a 44% increased risk of gestational diabetes mellitus in women who had a prior SAB. We did not find an association between SAB and the subsequent development of pre-eclampsia, however, women who experience RPL had increased odds of subsequently developing pre-eclampsia. RPL was also not associated with subsequent development of gestational diabetes mellitus, gestational hypertension, with no studies reporting on an association with non-pregnancy related diabetic or hypertensive disorders. There was evidence to suggest women who experience a prior SAB were at had increased odds of subsequently developing gestational hypertension, although the number of studies include in the meta-analysis was small (n = 3). There was no evidence to support an association between IAs and subsequent development of gestational diabetes mellitus, pre-eclampsia, gestational hypertension, and non-pregnancy related diabetes. Further, no study reported on the association between IA and non-pregnancy hypertension in later life.

Past reviews have attempted to elucidate the association between a prior early pregnancy loss and the subsequent development of cardiovascular disease,^77^, ^78^, ^79^ without attention to intermediary factors, such as diabetic and hypertensive disorders. This review undertook a comprehensive review of the findings from observational studies that investigated the association between a prior early pregnancy loss and the subsequent risk of developing a diabetic or hypertensive disorders, some of which are modifiable and can thereby prevent the onset of cardiovascular disease. Consistent with the results of previous reviews,^12^^,^^13^ our findings indicated that women with a history of SAB had an increased risk of gestational diabetes mellitus. A recent meta-analysis investigated the association between early pregnancy loss and gestational diabetes mellitus; however, despite the underlying characteristics of women with SAB and IA varied, this study failed to differentiate between SAB and IA, which is an important factor as there may be baseline differences between women who experience a SAB and women who elected to have an IA. This may have contributed to the authors finding that women who had an early pregnancy loss were at an increased risk of gestational diabetes mellitus compared to those in our review, in which women who experienced a prior SAB had increased odds of developing gestational diabetes mellitus but there was no association when IA was the exposure. Our findings were similar to one other review^12^ that investigated the association between a prior SAB and the risk of developing either gestational diabetes mellitus or a non-pregnancy diabetic disorder; however they did include IA as an exposure. Unlike other studies, our study evaluated the risks of the subsequent development of gestational diabetes mellitus by separately by previous SAB, IA and RPL, to provide more context in the interpretation of the effect of early pregnancy loss on gestational diabetes mellitus.

This was the first meta-analysis investigating the association between early pregnancy loss and the subsequent development of hypertensive disorders in pregnancy, thus direct comparison with previous studies is not possible. Our meta-analysis found no association between SAB and pre-eclampsia, with four of the included studies^52^^,^^54^^,^^59^^,^^72^ reporting SAB as a protective factor to subsequent pre-eclampsia, while three studies^46^^,^^57^^,^^63^ reported a strong association. There was no obvious distinction in study design, sample populations, country-level income or risk of bias that differentiated the results among the included studies. Although the exact biological mechanisms that link early pregnancy loss to a future maternal risk of a of hypertension disorders are difficult to elucidate; however, it is likely due to a complex interplay between genetics, endothelial dysfunction and metabolic syndrome^80^ that could differentiate between maternal populations. Strengthening the evidence for this genetic link is that women who have a history of RPL prior to their first birth have a higher risk of coronary heart disease, which is both diabetic and hypertensive disorders are a precursor to cardiovascular disease.^81^ This is supported by our finding that women who experienced a RPL had increased odds of developing pre-eclampsia in their subsequent pregnancy, thus further clinical investigation of the underlying causes of SABs may help prevent women progressing to RPLs and thus reduce any potential risk for subsequent development of diabetic and hypertensive disorders. Further evidence to support a link between early pregnancy loss with hypertensive disorders includes a susceptibility to metabolic syndrome across families, where this condition manifests itself in a combination of high blood glucose, hypertension, obesity and dyslipidaemia.^82^ Placental dysfunctions triggered by endothelial dysfunction underlies adverse pregnancy outcomes such as pre-eclampsia, gestational diabetes mellitus, and SAB.^83^ Recent research has indicated that endothelial dysfunction persists for several years beyond pregnancy, leading to an increased risk of the subsequent development of type 2 diabetes and hypertension disorders beyond pregnancy.^84^

This review included a large body of evidence from 22 countries. However, there are number of limitations. First, the majority of the included studies relied on a self-report of early pregnancy loss. Women are more likely to report and accurately recall a life-changing event such as early pregnancy loss; however, there may have been some underreporting of very early pregnancy losses (<5–6 gestational weeks), partly due to events that may not have been recognised. A barrier in research investigating the associations between early pregnancy losses and subsequent complications in pregnancy and beyond, is a lack of accurate data on early pregnancy loss.^11^ To accurately capture all early pregnancy losses, a study would be required to prospectively follow women from the time of conception. However, such studies are generally deemed unfeasible. A second limitation of this review and the included studies is that we were not able to capture the subsequent risk of a pregnancy related diabetic or hypertensive disorder in women who never achieved a pregnancy. A third limitation is that included studies that did not screen for women with disorders (such as thrombophilia or antiphospholipid syndrome), which may have increased the risk of experience a SAB. It is plausible that these disorders may have confounded the association between SAB and the subsequent development of diabetic and hypertensive disorder during subsequent pregnancies. Fourth, we were unable to undertake a meta-analysis for all exposure-outcome associations, in particular there was a too few studies examining associations between early pregnancy loss and the subsequent development to nonpregnancy hypertension. The meta-analysis included in this study reported significant heterogeneity between the studies. Particularly, a substantial heterogeneity observed for the association between SAB and gestational diabetes mellitus, which may be attributed to changes in screening protocols and diagnostic criteria between studies and countries. Additionally, variations in the adjustment for confounding variables, study design and sample size across studies may have contributed to the heterogeneity. When the outcome was gestational diabetes mellitus, we undertook a meta-regression by year of publication to accommodate for change in diagnosis protocols over-time. Finally, we limited our included studies to English language publications.

Early pregnancy loss can have long-term health consequences in the subsequent pregnancy and beyond. The findings of this review found that women with a history of SAB are at risk for subsequent development of gestational diabetes mellitus. Women with a history of RPL have increased odds of developing a pre-eclampsia in a subsequent pregnancy; however, this risk was not evident for women who had experience one SAB. This study found no evidence of an association between IA and the subsequent development of pregnancy and non-pregnancy diabetic and hypertensive disorders. Future research should be undertaken to determine potential associations between early pregnancy loss events and the subsequent development of non-pregnancy related diabetes and hypertensive disorders in later life.

## Contributors

DF, GAT, and GP conceived and designed the study. JD, SDN, BAD, GAT undertook the data analysis and verified the underlying data. JD drafted the initial version of the manuscript. All authors contributed to the interpretation of the data and critically revised the manuscript. All authors had full access to tables and figures in the study and can take responsibility for the integrity of the data and the accuracy of the data analysis. All authors read and approved the final version of the manuscript. The corresponding author attests that all listed authors meet authorship criteria.

## Data sharing statement

The datasets generated and/or analysed during the current study are available in Supplementary materials. Rough data supporting reported results are available from the corresponding author on reasonable request.

## Declaration of interests

The authors have no conflicts of interest to declare.

## References

[1] Sedgh G.D., Bearak J.P., Singh S.P. (2016). Abortion incidence between 1990 and 2014: global, regional, and subregional levels and trends. Lancet.

[2] Mohangoo A.D., Blondel B., Gissler M. (2013). International comparisons of fetal and neonatal mortality rates in high-income countries: should exclusion thresholds be based on birth weight or gestational age?. PLoS One.

[3] Christou A., Dibley M.J., Raynes-Greenow C. (2017). Beyond counting stillbirths to understanding their determinants in low- and middle-income countries: a systematic assessment of stillbirth data availability in household surveys. Trop Med Int Health.

[4] Dimitriadis E., Menkhorst E., Saito S., Kutteh W.H., Brosens J.J. (2020). Recurrent pregnancy loss. Nat Rev Dis Prim.

[5] Bearak J., Popinchalk A., Ganatra B. (2020). Unintended pregnancy and abortion by income, region, and the legal status of abortion: estimates from a comprehensive model for 1990–2019. Lancet Global Health.

[6] Arnett D.K., Blumenthal R.S., Albert M.A. (2019). 2019 ACC/AHA guideline on the primary prevention of cardiovascular disease: a report of the American College of Cardiology/American Heart Association Task Force on clinical practice guidelines. Circulation.

[7] Piepoli M.F., Hoes A.W., Agewall S. (2016). 2016 European guidelines on cardiovascular disease prevention in clinical practice: the Sixth joint task force of the European society of cardiology and other societies on cardiovascular disease prevention in clinical practice (constituted by representatives of 10 societies and by invited experts) developed with the special contribution of the European association for cardiovascular prevention & rehabilitation (EACPR). Atherosclerosis.

[8] Wen Lo C.C., Lo A.C.Q., Leow S.H. (2020). Future cardiovascular disease risk for women with gestational hypertension: a systematic review and meta-analysis. J Am Heart Assoc.

[9] Wu P., Haththotuwa R., Kwok C.S. (2017). Preeclampsia and future cardiovascular Health: a systematic review and meta-analysis. Circ Cardiovasc Qual Outcomes.

[10] Kramer C.K., Campbell S., Retnakaran R. (2019). Gestational diabetes and the risk of cardiovascular disease in women: a systematic review and meta-analysis. Diabetologia.

[11] Quenby S., Gallos I.D., Dhillon-Smith R.K. (2021). Miscarriage matters: the epidemiological, physical, psychological, and economic costs of early pregnancy loss. Lancet.

[12] You Q., Jiang Q., Shani I. (2023). Miscarriage, stillbirth and the risk of diabetes in women: a systematic review and meta-analysis. Diabetes Res Clin Pract.

[13] Wang H., Guo X., Song Q. (2022). Association between the history of abortion and gestational diabetes mellitus: a meta-analysis. Endocrine.

[14] Okoth K., Subramanian A., Chandan J.S. (2022). Long term miscarriage-related hypertension and diabetes mellitus. Evidence from a United Kingdom population-based cohort study. PLoS One.

[15] Egerup P., Mikkelsen A.P., Kolte A.M. (2020). Pregnancy loss is associated with type 2 diabetes: a nationwide case–control study. Diabetologia.

[16] Kharazmi E., Lukanova A., Teucher B., Groß M.-L., Kaaks R. (2012). Does pregnancy or pregnancy loss increase later maternal risk of diabetes?. Eur J Epidemiol.

[17] Zhao Y., Zhao Y., Fan K., Jin L. (2022). Association of history of spontaneous or induced abortion with subsequent risk of gestational diabetes. JAMA Netw Open.

[18] Horn J., Tanz L.J., Stuart J.J. (2019). Early or late pregnancy loss and development of clinical cardiovascular disease risk factors: a prospective cohort study. BJOG.

[19] Leon B.M., Maddox T.M. (2015). Diabetes and cardiovascular disease: Epidemiology, biological mechanisms, treatment recommendations and future research. World J Diabetes.

[20] Ahmed S.K., Mahmood N., Malalla Z.H., Alsobyani F.M., Al-Kiyumi I.S., Almawi W.Y. (2015). C-reactive protein gene variants associated with recurrent pregnancy loss independent of CRP serum levels: a case-control study. Gene.

[21] Duhig K., Chappell L.C., Shennan A.H. (2016). Oxidative stress in pregnancy and reproduction. Obstet Med.

[22] Moher D., Liberati A., Tetzlaff J., Altman D.G. (2009). Reprint—preferred reporting items for systematic reviews and meta-analyses: the PRISMA statement. Phys Ther.

[23] Brooke B.S., Schwartz T.A., Pawlik T.M. (2021). MOOSE reporting guidelines for meta-analyses of observational studies. JAMA Surg.

[24] Foo D., Dunne J., Pereira G., Gebremedhin A., Duko B., Tessema G.A. (2022). Diabetic and hypertensive disorders following miscarriage: a protocol for systematic review and meta-analysis. Int J Environ Res Publ Health.

[25] ROBINS-E Development Group, Higgins J.M.R., Rooney A. (2024). http://www.riskofbias.info/welcome/robins-e-tool.

[26] Sterne J.A.C., Hernán M.A., Reeves B.C. (2016). ROBINS-I: a tool for assessing risk of bias in non-randomised studies of interventions. BMJ.

[27] Riley R.D., Higgins J.P.T., Deeks J.J. (2011). Interpretation of random effects meta-analyses. Br Med J.

[28] Higgins J.P.T.T.J., Chandler J., Cumpston M. (2019). http://www.training.cochrane.org/handbook.

[29] Abi-Said D., Annegers J.F., Combs-Cantrell D., Frankowski R.F., Willmore L.J. (1995). Case-control study of the risk factors for eclampsia. Am J Epidemiol.

[30] Bhat M., Ramesha K.N., Sarma S.P., Menon S., Sowmini C.V., Ganesh Kumar S. (2010). Determinants of gestational diabetes mellitus: a case control study in a district tertiary care hospital in south India. Int J Diabetes Dev Ctries.

[31] Cozzolino M., Rizzello F., Riviello C., Romanelli C., Coccia Elisabetta M. (2019). Ongoing pregnancies in patients with unexplained recurrent pregnancy loss: adverse obstetric outcomes. Hum Fertil.

[32] Eras J.L., Saftlas A.F., Triche E., Hsu C.-D., Risch H.A., Bracken M.B. (2000). Abortion and its effect on risk of preeclampsia and transient hypertension. Epidemiology.

[33] Liu L.-Y., Zhang Y.-L., Li L. (2017). Risk factor of gestational diabetes among healthy Chinese women: an observational study. Biomed Res.

[34] Parker S.E., Gissler M., Ananth C.V., Werler M.M. (2014). Induced abortions and the risk of preeclampsia among nulliparous women. Am J Epidemiol.

[35] Sembiring R.L., Mappaware N.A., Usman A.N. (2019). Relationship between characteristics and obstetric history with hypertension in pregnancy. Enferm Clin.

[36] Sheiner E., Levy A., Katz M., Mazor M. (2005). Pregnancy outcome following recurrent spontaneous abortions. Eur J Obstet Gynecol Reprod Biol.

[37] Stitterich N., Shepherd J., Koroma M.M., Theuring S. (2021). Risk factors for preeclampsia and eclampsia at a main referral maternity hospital in Freetown, Sierra Leone: a case-control study. BMC Pregnancy Childbirth.

[38] Su Y.-Y., Zhang J.-Z., Wang F. (2017). Risk factors and adverse outcomes of preeclampsia: a tertiary care centrebased study in China. Biomed Res.

[39] Unnikrishnan B., Rathi P., Bhat S.K. (2020). Risk factors of gestational diabetes mellitus: a hospital-based pair-matched case-control study in coastal south India. S Afr J Obstet Gynaecol.

[40] Vanek M., Sheiner E., Levy A., Mazor M. (2004). Chronic hypertension and the risk for adverse pregnancy outcome after superimposed pre-eclampsia. Int J Gynaecol Obstet.

[41] Ali N., Elbarazi I., Ghazal-Aswad S. (2020). Impact of recurrent miscarriage on maternal outcomes in subsequent pregnancy: the mutaba’ah study. Int J Wom Health.

[42] Li T.C., Yip B.H.K., Chen X. (2023). Recurrent miscarriage and risk of obstetric and perinatal complications in subsequent pregnancy: abridged secondary publication. Hong Kong Med J.

[43] Ranthe M.F., Andersen E.A.W., Wohlfahrt J., Bundgaard H., Melbye M., Boyd H.A. (2013). Pregnancy loss and later risk of Atherosclerotic disease. Circulation.

[44] Taye H., Kabthymer R.H., Hailu S. (2022). Previous adverse pregnancy events as a predictor of gestational diabetes mellitus in Southern Ethiopia: a case control study. Article. Curr Med Res Opin.

[45] Ticconi C., Pietropolli A., Specchia M. (2020). Pregnancy-related complications in women with recurrent pregnancy loss: a prospective cohort study. J Clin Med.

[46] Weintraub A.Y.M.D., Sergienko R., Harlev A.M.D. (2011). An initial miscarriage is associated with adverse pregnancy outcomes in the following pregnancy. Am J Obstet Gynecol.

[47] AlKasseh A.S.M., Zaki N.M., Aljeesh Y.I., Soon L.K. (2014). Risk factors of gestational diabetes mellitus in the refugee population in Gaza Strip: a case-control study. East Mediterr Health J.

[48] Askari M., Dadbinpour A., Ekraminasab S., Shukohifar M. (2023). Incidence and risk factors related to gestational diabetes mellitus among women in Yazd: a prospective cohort study. World J Peri Neonatol.

[49] Feleke B.E. (2018). Determinants of gestational diabetes mellitus: a case-control study. J Matern Fetal Neonatal Med.

[50] Lao T.T., Hui A.S.Y., Law L.-W., Sahota D.S. (2018). Prior abortion history and pregnancy hypertensive disorders in primiparous gravidae. Pregnancy Hypertens.

[51] Christians J.K., Huicochea Munoz M.F. (2020). Pregnancy complications recur independently of maternal vascular malperfusion lesions. PLoS One.

[52] Eskenazi B., Fenster L., Sidney S. (1991). A multivariate analysis of risk factors for preeclampsia. JAMA.

[53] Huo Y., Cheng L., Wang C. (2021). Associations between parity, pregnancy loss, and breastfeeding duration and risk of maternal type 2 diabetes: an observational cohort study. J Diabetes.

[54] Mohamedain A., Rayis D.A., AlHabardi N., Adam I. (2022). Association between previous spontaneous abortion and preeclampsia: a case-control study. BMC Pregnancy Childbirth.

[55] Parazzini F., Bortolus R., Chatenoud L. (1996). Risk factors for pregnancy-induced hypertension in women at high risk for the condition. Epidemiology.

[56] Peters S.A.E., Yang L., Guo Y. (2019). Pregnancy, pregnancy loss and the risk of diabetes in Chinese women: findings from the China Kadoorie Biobank. Eur J Epidemiol.

[57] Sepidarkish M., Almasi-Hashiani A., Maroufizadeh S., Vesali S., Pirjani R., Samani R.O. (2017). Association between previous spontaneous abortion and pre-eclampsia during a subsequent pregnancy. Int J Gynaecol Obstet.

[58] Sibai B.M., Ewell M., Levine R.J. (1997). Risk factors associated with preeclampsia in healthy nulliparous women. Am J Obstet Gynecol.

[59] Sun H., Mao J., Su X., Du Q. (2023). Impact of spontaneous abortion history and induced abortion history on perinatal outcomes of singleton pregnancies. BMC Publ Health.

[60] Vaajala M., Liukkonen R., Ponkilainen V., Kekki M., Mattila V.M., Kuitunen I. (2023). Previous induced abortion or miscarriage is associated with increased odds for gestational diabetes: a nationwide register-based cohort study in Finland. Acta Diabetol.

[61] Yang Q., Song C., Jiang J. (2018). Association of reproductive history with hypertension and prehypertension in Chinese postmenopausal women: a population-based cross-sectional study. Hypertens Res.

[62] Ausbeck E.B., Blanchard C.T., Neely C., Tita A.T., Szychowski J.M., Harper L.M. (2021).

[63] Bhattacharya S., Townend J., Shetty A., Campbell D., Bhattacharya S. (2008). Does miscarriage in an initial pregnancy lead to adverse obstetric and perinatal outcomes in the next continuing pregnancy?. BJOG.

[64] Gunnarsdottir J.M.D., Stephansson O.M.D.P., Cnattingius S.M.D.P., Åkerud H.M.D.P., Wikström A.-K.M.D.P. (2014). Risk of placental dysfunction disorders after prior miscarriages: a population-based study. Am J Obstet Gynecol.

[65] Makhlouf M.A., Clifton R.G., Roberts J.M. (2014). Adverse pregnancy outcomes among women with prior spontaneous or induced abortions. Am J Perinatol.

[66] Olayemi O., Strobino D., Adedapo K., Aimakhu C., Odukogbe A.-T., Salako B. (2010). Influence of previous abortions and new paternity on the risk of hypertension in nulliparous parturients in Ibadan: a cohort study: abortion and hypertension in pregnancy in Nigeria. J Obstet Gynaecol Res.

[67] Roepke E.R., Christiansen O.B., Källén K., Hansson S.R. (2021). Women with a history of recurrent pregnancy loss are a high-risk population for adverse obstetrical outcome: a retrospective cohort study. J Clin Med.

[68] Saftlas A.F., Levine R.J., Klebanoff M.A. (2003). Abortion, changed paternity, and risk of preeclampsia in nulliparous women. Am J Epidemiol.

[69] Su Y., Xie X., Zhou Y. (2020). Association of induced abortion with hypertensive disorders of pregnancy risk among nulliparous women in China: a prospective cohort study. Sci Rep.

[70] Trogstad L., Magnus P., Moffett A., Stoltenberg C. (2009). The effect of recurrent miscarriage and infertility on the risk of pre-eclampsia. BJOG.

[71] Trogstad L., Magnus P., Skjærven R., Stoltenberg C. (2008). Previous abortions and risk of pre-eclampsia. Int J Epidemiol.

[72] Xiong X., Fraser W.D., Demianczuk N.N. (2002). History of abortion, preterm, term birth, and risk of preeclampsia: a population-based study. Am J Obstet Gynecol.

[73] Yang H., Wei Y., Gao X. (2009). Risk factors for gestational diabetes mellitus in Chinese women-a prospective study of 16286 pregnant women in China. Diabet Med.

[74] Zhang J., Liu X., Rao L. (2023). Adverse obstetric and perinatal outcomes of patients with history of recurrent miscarriage: a retrospective cohort study. Fertil Steril.

[75] Zhang Y., Xiao C.-M., Zhang Y. (2021). Factors associated with gestational diabetes mellitus: a meta-analysis. J Diabetes Res.

[76] Liu B., Song L., Li H. (2018). History of spontaneous miscarriage and the risk of diabetes mellitus among middle-aged and older Chinese women. Acta Diabetol.

[77] Grandi S.M., Filion K.B., Yoon S. (2019). Cardiovascular disease-related morbidity and mortality in women with a wistory of wregnancy womplications: systematic weview and neta-analysis. Circulation.

[78] Kyriacou H., Al-Mohammad A., Muehlschlegel C. (2022). The risk of cardiovascular diseases after miscarriage, stillbirth, and induced abortion: a systematic review and meta-analysis. Eur Heart J Open.

[79] Okoth K., Chandan J.S., Marshall T. (2020). Association between the reproductive health of young women and cardiovascular disease in later life: umbrella review. BMJ.

[80] Zhu X.-Z., Deng Z.-M., Dai F.-F., Liu H., Cheng Y.-X. (2023). The impact of early pregnancy metabolic disorders on pregnancy outcome and the specific mechanism. Eur J Med Res.

[81] Mahendru A.A., Everett T.R., McEniery C.M., Wilkinson I.B., Lees C.C. (2013). Cardiovascular function in women with recurrent miscarriage, pre-eclampsia and/or intrauterine growth restriction. J Matern Fetal Neonatal Med.

[82] Murphy E. (2015). Pregnancy in women with inherited metabolic disease. Obstet Med.

[83] McElwain C.J., Tuboly E., McCarthy F.P., McCarthy C.M. (2020). Mechanisms of endothelial dysfunction in pre-eclampsia and gestational diabetes mellitus: windows into future cardiometabolic health?. Front Endocrinol.

[84] Germain A.M., Romanik M.C., Guerra I. (2007). Endothelial dysfunction: a link among preeclampsia, recurrent pregnancy loss, and future cardiovascular events?. Hypertension.

