# Supplementary material for: Diabetic and hypertensive disorders following early pregnancy loss: a systematic review and meta-analysis
Source: eClinicalMedicine. 2024 Mar 27;71:102560. doi: 10.1016/j.eclinm.2024.102560 (PMC11133813; doi:10.1016/j.eclinm.2024.102560)
Supplement: Supplementary Figures and Tables [file mmc1.docx]

**Diabetic and hypertensive disorders following early pregnancy loss: a systematic review and meta-analysis**

**Authors:** Jennifer Dunne, Damien Foo, Berihun A Dachew, Bereket Duko, Amanuel T Gebremedhin, Sylvester D Nyadanu, Gavin Pereira, Gizachew A Tessema

**Table of Contents**

[Supplementary Table 1. Search strategy for systematic search of the literature 2](#_Toc159179455)

[Supplementary Table 2. Preferred Reporting for Systematic Review and Meta-Analysis (PRISMA) 2020 completed checklist 4](#_Toc159179456)

[Supplementary Table 3. Meta-analyses of Observational Studies in Epidemiology (MOOSE) completed checklist 6](#_Toc159179457)

[Supplementary Table 4. Records excluded at full-text screening with reasons (n=15) 8](#_Toc159179458)

[Supplementary Table 5. Characteristics of the included studies (n=52) 9](#_Toc159179459)

[Supplementary Table 6: Risk of bias assessment of the included studies based on the Risk of Bias in Non-randomized Studies of Exposure (ROBINS-E) tool 16](#_Toc159179460)

[Supplementary Table 7. Confounders included in the analysis of the association between early pregnancy loss and the development of a diabetic or a hypertensive disorder 18](#_Toc159179461)

[Supplementary Figure 1. Meta-analysis of the association between a prior induced abortion and the risk of developing gestational diabetes mellitus in a subsequent pregnancy. 21](#_Toc159179462)

[Supplementary Figure 2. Meta-analysis of the association between a recurrent pregnancy loss and the risk of developing gestational diabetes mellitus in a subsequent pregnancy 22](#_Toc159179463)

[Supplementary Figure 3. Meta-analysis of the association between a spontaneous abortion and the risk of developing non-pregnancy related diabetes. 23](#_Toc159179464)

[Supplementary Figure 4. Meta-analysis of the association between an induced abortion and the risk of developing non-pregnancy related diabetes. 24](#_Toc159179465)

[25](#_Toc159179466)

[Supplementary Figure 5. Meta-analysis of the association between an induced abortion and the risk of developing pre-eclampsia in a subsequent pregnancy. 25](#_Toc159179467)

[Supplementary Figure 6. Meta-analysis of the association between a recurrent pregnancy loss and the risk of developing pre-eclampsia in a subsequent pregnancy. 26](#_Toc159179468)

[Supplementary Figure 7. Meta-analysis of the association between a spontaneous abortion and the risk of developing gestational hypertension in a subsequent pregnancy. 27](#_Toc159179469)

[Supplementary Figure 8. Meta-analysis of the association between an induced abortion and the risk of developing gestational hypertension in a subsequent pregnancy. 28](#_Toc159179470)

[Supplementary Figure 9. Meta-analysis of the association between an induced abortion and the risk of developing non-pregnancy hypertension. 29](#_Toc159179471)

[Supplementary Figure 10. Funnel plot with pseudo 95% confidence limits for the odds ratio of the association between a prior spontaneous abortion and the risk of developing gestational diabetes mellitus from the nine included studies. 30](#_Toc159179472)

[Supplementary Figure 11. Funnel plot with pseudo 95% confidence limits for the odds ratio of the association between a prior spontaneous abortion and the risk of developing pre-eclampsia from the 12 included studies. 31](#_Toc159179473)

[References of the included studies (n=52) 32](#_Toc159179474)

**Supplementary Table 1**. Search strategy for systematic search of the literature

| **Search engine** | **Search string** |
| --- | --- |
| CINAHL Plus | MH "Abortion, Spontaneous+" OR MH "Abortion, Induced+" + (TI ("SAB" OR "Habitual SAB" OR "Recurrent SAB" OR "Spontaneous abortion" OR "IA" OR "Habitual abortion" OR "Recurrent abortion" OR "Incomplete Abortion" OR "Spontaneous loss" OR "Pregnancy loss" OR "Infant loss" or "Fetal loss" OR "Foetal loss" OR "Fetus loss" OR "Foetus loss" OR "Fetal death" OR "Foetal death" OR "Fetus death" OR "Foetus death" OR "Fetal demise" OR "Foetal demise" OR "Fetus demise" OR "Foetus demise")) OR (AB ("SAB" OR "Habitual SAB" OR "Recurrent SAB" OR "Spontaneous abortion" OR "IA" OR "Habitual abortion" OR "Recurrent abortion" OR "Incomplete Abortion" OR "Spontaneous loss" OR "Pregnancy loss" OR "Infant loss" or "Fetal loss" OR "Foetal loss" OR "Fetus loss" OR "Foetus loss" OR "Fetal death" OR "Foetal death" OR "Fetus death" OR "Foetus death" OR "Fetal demise" OR "Foetal demise" OR "Fetus demise" OR "Foetus demise")) + MH "Diabetes Mellitus+" + (TI ("Diabetes" OR "Diabetes mellitus" "Type 1 diabetes" OR "Type-1 diabetes" OR "Type 1 diabetes mellitus" OR "Type-1 diabetes mellitus" OR "Type 2 diabetes" OR "Type-2 diabetes" OR "Type 2 diabetes mellitus" OR "Type-2 diabetes mellitus")) OR (AB ("Diabetes" OR "Diabetes mellitus" OR "Type 1 diabetes" OR "Type-1 diabetes" OR "Type 1 diabetes mellitus" OR "Type-1 diabetes mellitus" OR "Type 2 diabetes" OR "Type-2 diabetes" OR "Type 2 diabetes mellitus" OR "Type-2 diabetes mellitus")) + MH "Pregnancy in Diabetes+" + (TI ("Hyperten*" OR ("High" N2 "Blood pressure") OR ("Increas*" N2 "Blood pressure") OR ("Elevat*" N2 "Blood pressure"))) OR (AB ("Hyperten*" OR ("High" N2 "Blood pressure") OR ("Increas*" N2 "Blood pressure") OR ("Elevat*" N2 "Blood pressure"))) OR (AB ("Hyperten*" OR ("High" N2 "Blood pressure") OR ("Increas*" N2 "Blood pressure") OR ("Elevat*" N2 "Blood pressure"))) OR (AB ("Hyperten*" OR ("High" N2 "Blood pressure") OR ("Increas*" N2 "Blood pressure") OR ("Elevat*" N2 "Blood pressure"))) + MH "Pregnancy-Induced Hypertension+" |
| Ovid (Embase/Medline) | ("SAB" or "Habitual SAB" or "Recurrent SAB" or "Spontaneous abortion" or "IA" or "Habitual abortion" or "Recurrent abortion" or "Incomplete Abortion" or "Spontaneous loss" or "Pregnancy loss" or "Infant loss" or "Fetal loss" or "Foetal loss" or "Fetus loss" or "Foetus loss" or "Fetal death" or "Foetal death" or "Fetus death" or "Foetus death" or "Fetal demise" or "Foetal demise" or "Fetus demise" or "Foetus demise").ti,ab AND ("Diabetes" or "Diabetes mellitus" or "Type 1 diabetes" or "Type-1 diabetes" or "Type 1 diabetes mellitus" or "Type-1 diabetes mellitus" or "Type 2 diabetes" or "Type-2 diabetes" or "Type 2 diabetes mellitus" or "Type-2 diabetes mellitus").ti,ab AND (("Diabetes" or "Diabetes mellitus") adj3 ("Gestation*" or "Pregnan*")).ti,ab AND ("Hyperten*" or (("High" or "Increas*" or "Elevat*") adj3 ("Blood pressure"))).ti,ab |
| Proquest | (MESH("Abortion, Spontaneous" or "Abortion, Induced" or "Fetal Death") OR TI,AB("SAB" or "Habitual SAB" or "Recurrent SAB" or "Spontaneous abortion" or "IA" or "Habitual abortion" or "Recurrent abortion" or "Incomplete Abortion" or "Spontaneous loss" or "Pregnancy loss" or "Infant loss" or "Fetal loss" or "Foetal loss" or "Fetus loss" or "Foetus loss" or "Fetal death" or "Foetal death" or "Fetus death" or "Foetus death" or "Fetal demise" or "Foetal demise" or "Fetus demise" or "Foetus demise")) AND (MESH("Diabetes Mellitus") OR TI,AB("Diabetes" or "Diabetes mellitus" or "Type 1 diabetes" or "Type-1 diabetes" or "Type 1 diabetes mellitus" or "Type-1 diabetes mellitus" or "Type 2 diabetes" or "Type-2 diabetes" or "Type 2 diabetes mellitus" or "Type-2 diabetes mellitus").ti,ab) AND (MESH("Diabetes, Gestational" or "Pregnancy in Diabetics") OR TI,AB(("Diabetes" or "Diabetes mellitus") NEAR/2 ("Gestation*" or "Pregnan*"))) AND (MESH("Hypertension") OR TI,AB("Hypertension" or (("High" or "Increas*" or "Elevat*") NEAR/2 "Blood pressure"))) AND (MESH("Hypertension, Pregnancy-Induced") OR TI,AB(("Hyperten*" or ("High" NEAR/2 "Blood pressure") or ("Increas*" NEAR/2 "Blood pressure") or ("Elevat*" NEAR/2 "Blood pressure")) NEAR/2 ("Gestation*" or "Pregnan*") or "Eclampsia" or "Preeclampsia" or "Pre-eclampsia" or "Pre eclampsia")) |
| PubMed | ("Abortion, Spontaneous"[MeSH Terms] or "Abortion, Induced"[MeSH Terms] or "Fetal Death"[MeSH Terms] OR "SAB"[TiAb] or "Habitual SAB"[TiAb] or "Recurrent SAB"[TiAb] or "Spontaneous abortion"[TiAb] or "IA"[TiAb] or "Habitual abortion"[TiAb] or "Recurrent abortion"[TiAb] or "Incomplete Abortion"[TiAb] or "Spontaneous loss"[TiAb] or "Pregnancy loss"[TiAb] or "Infant loss"[TiAb] or "Fetal loss"[TiAb] or "Foetal loss"[TiAb] or "Fetus loss"[TiAb] or "Foetus loss"[TiAb] or "Fetal death"[TiAb] or "Foetal death"[TiAb] or "Fetus death"[TiAb] or "Foetus death"[TiAb] or "Fetal demise"[TiAb] or "Foetal demise"[TiAb] or "Fetus demise"[TiAb] or "Foetus demise"[TiAb]) AND ("Diabetes Mellitus"[MeSH Terms] OR "Diabetes"[TiAb] or "Diabetes mellitus"[TiAb] or "Type 1 diabetes"[TiAb] or "Type-1 diabetes"[TiAb] or "Type 1 diabetes mellitus"[TiAb] or "Type-1 diabetes mellitus"[TiAb] or "Type 2 diabetes"[TiAb] or "Type-2 diabetes"[TiAb] or "Type 2 diabetes mellitus"[TiAb] or "Type-2 diabetes mellitus"[TiAb]) AND ("Diabetes, Gestational"[MeSH Terms] or "Pregnancy in Diabetics"[MeSH Terms]) OR ("Diabetes"[TiAb] or "Diabetes mellitus"[TiAb]) and ("Gestation*"[TiAb] or "Pregnan*"[TiAb])) AND ("Hypertension"[MeSH Terms] OR ("High"[TiAb] or "Increas*"[TiAb] or "Elevat*"[TiAb]) and "Blood pressure"[TiAb]) AND ("Hypertension, Pregnancy-Induced"[MeSH Terms] OR (("Hyperten*"[TiAb] or (("High"[TiAb] or "Increas*"[TiAb] or "Elevat*"[TiAb]) and "Blood pressure"[TiAb]))) and ("Gestation*"[TiAb] or "Pregnan*"[TiAb])) |
| Scopus | TITLE-ABS-KEY ( ( "SAB" OR "Habitual SAB" OR "Recurrent SAB" OR "Spontaneous abortion" OR "IA" OR "Habitual abortion" OR "Recurrent abortion" OR "Incomplete Abortion" OR "Spontaneous loss" OR "Pregnancy loss" OR "Infant loss" OR "Fetal loss" OR "Foetal loss" OR "Fetus loss" OR "Foetus loss" OR "Fetal death" OR "Foetal death" OR "Fetus death" OR "Foetus death" OR "Fetal demise" OR "Foetal demise" OR "Fetus demise" OR "Foetus demise" ) ) AND TITLE-ABS-KEY ( ( "Diabetes" OR "Diabetes mellitus" OR "Type 1 diabetes" OR "Type-1 diabetes" OR "Type 1 diabetes mellitus" OR "Type-1 diabetes mellitus" OR "Type 2 diabetes" OR "Type-2 diabetes" OR "Type 2 diabetes mellitus" OR "Type-2 diabetes mellitus" ) ) AND TITLE-ABS-KEY ( ( "Diabetes" OR "Diabetes mellitus" ) W/2 ( "Gestation*" OR "Pregnan*" ) ) AND TITLE-ABS-KEY ( "Hyperten*" ) OR TITLE-ABS-KEY ( ( "High" OR "Increas*" OR "Elevat*" ) W/2 ( "Blood pressure" ) ) AND ( TITLE-ABS-KEY ( ( "Hyperten*" ) W/2 ( "Gestation*" OR "Pregnan*" ) ) ) OR ( ( TITLE-ABS-KEY ( ( "High" OR "Increas*" OR "Elevat*" ) W/2 ( "Blood pressure" ) ) ) AND ( TITLE-ABS-KEY ( "Gestation*" OR "Pregnan*" ) ) ) |
| Web of Science | ("SAB" or "Habitual SAB" or "Recurrent SAB" or "Spontaneous abortion" or "IA" or "Habitual abortion" or "Recurrent abortion" or "Incomplete Abortion" or "Spontaneous loss" or "Pregnancy loss" or "Infant loss" or "Fetal loss" or "Foetal loss" or "Fetus loss" or "Foetus loss" or "Fetal death" or "Foetal death" or "Fetus death" or "Foetus death" or "Fetal demise" or "Foetal demise" or "Fetus demise" or "Foetus demise") AND ("Diabetes" or "Diabetes mellitus" or "Type 1 diabetes" or "Type-1 diabetes" or "Type 1 diabetes mellitus" or "Type-1 diabetes mellitus" or "Type 2 diabetes" or "Type-2 diabetes" or "Type 2 diabetes mellitus" or "Type-2 diabetes mellitus") AND (Hyperten*) OR ("High" or "Increas*" or "Elevat*") NEAR/2 ("Blood pressure") AND ((("Hyperten*") NEAR ("Gestation" or "Pregnan*")) OR (("High" or "Increas*" or "Elevat*") NEAR/2 ("Blood pressure")) NEAR/2 ("Gestation" or "Pregnan")) |
| Google Scholar | (SAB\|"Spontaneous\|Induced\|Habitual\|Recurrent\|Incomplete(Abortion)"\|"Spontaneous\|Pregnancy\|Infant\|Fetal(Loss)"\|"Fetal(Death\|Demise)") and (Diabetes\|Hypertension\|"High\|increased\|elevated(blood pressure)"\|Eclampsia) |

Supplementary Table 2. Preferred Reporting for Systematic Review and Meta-Analysis (PRISMA) 2020 completed checklist

| **Section and Topic** | **Item #** | **Checklist item** | **Location where item is reported** |
| --- | --- | --- | --- |
| **TITLE** | | |  |
| Title | 1 | Identify the report as a systematic review. | Pg 1, line 1 |
| **ABSTRACT** | | |  |
| Abstract | 2 | See the PRISMA 2020 for Abstracts checklist. | Pg 2, line 1 |
| **INTRODUCTION** | | |  |
| Rationale | 3 | Describe the rationale for the review in the context of existing knowledge. | Pg 4, line 1 |
| Objectives | 4 | Provide an explicit statement of the objective(s) or question(s) the review addresses. | Pg 4, line 39 |
| **METHODS** | | |  |
| Eligibility criteria | 5 | Specify the inclusion and exclusion criteria for the review and how studies were grouped for the syntheses. | Pg 5, line 17 |
| Information sources | 6 | Specify all databases, registers, websites, organisations, reference lists and other sources searched or consulted to identify studies. Specify the date when each source was last searched or consulted. | Pg 5, line 1 |
| Search strategy | 7 | Present the full search strategies for all databases, registers and websites, including any filters and limits used. | Supp T S1 |
| Selection process | 8 | Specify the methods used to decide whether a study met the inclusion criteria of the review, including how many reviewers screened each record and each report retrieved, whether they worked independently, and if applicable, details of automation tools used in the process. | Pg 5, line 28 |
| Data collection process | 9 | Specify the methods used to collect data from reports, including how many reviewers collected data from each report, whether they worked independently, any processes for obtaining or confirming data from study investigators, and if applicable, details of automation tools used in the process. | Pg 5, line 29 |
| Data items | 10a | List and define all outcomes for which data were sought. Specify whether all results that were compatible with each outcome domain in each study were sought (e.g. for all measures, time points, analyses), and if not, the methods used to decide which results to collect. | Pg 5, line 20 |
|  | 10b | List and define all other variables for which data were sought (e.g. participant and intervention characteristics, funding sources). Describe any assumptions made about any missing or unclear information. | Pg 5, line 38 |
| Study risk of bias assessment | 11 | Specify the methods used to assess risk of bias in the included studies, including details of the tool(s) used, how many reviewers assessed each study and whether they worked independently, and if applicable, details of automation tools used in the process. | Pg 5, line 43 |
| Effect measures | 12 | Specify for each outcome the effect measure(s) (e.g. risk ratio, mean difference) used in the synthesis or presentation of results. | Pg 6, line 15 |
| Synthesis methods | 13a | Describe the processes used to decide which studies were eligible for each synthesis (e.g. tabulating the study intervention characteristics and comparing against the planned groups for each synthesis (item #5)). | Pg 6, line 11 |
|  | 13b | Describe any methods required to prepare the data for presentation or synthesis, such as handling of missing summary statistics, or data conversions. |  |
|  | 13c | Describe any methods used to tabulate or visually display results of individual studies and syntheses. | Pg 6, line 10 |
|  | 13d | Describe any methods used to synthesize results and provide a rationale for the choice(s). If meta-analysis was performed, describe the model(s), method(s) to identify the presence and extent of statistical heterogeneity, and software package(s) used. | Pg 6, line 17 |
|  | 13e | Describe any methods used to explore possible causes of heterogeneity among study results (e.g. subgroup analysis, meta-regression). | Pg 6, line 25 |
|  | 13f | Describe any sensitivity analyses conducted to assess robustness of the synthesized results. | Pg 6, line 28 |
| Reporting bias assessment | 14 | Describe any methods used to assess risk of bias due to missing results in a synthesis (arising from reporting biases). | NA |
| Certainty assessment | 15 | Describe any methods used to assess certainty (or confidence) in the body of evidence for an outcome. |  |
| **RESULTS** | | |  |
| Study selection | 16a | Describe the results of the search and selection process, from the number of records identified in the search to the number of studies included in the review, ideally using a flow diagram. | Figure 1 |
|  | 16b | Cite studies that might appear to meet the inclusion criteria, but which were excluded, and explain why they were excluded. | Supp T S2 |
| Study characteristics | 17 | Cite each included study and present its characteristics. | Supp T S1 |
| Risk of bias in studies | 18 | Present assessments of risk of bias for each included study. |  |
| Results of individual studies | 19 | For all outcomes, present, for each study: (a) summary statistics for each group (where appropriate) and (b) an effect estimate and its precision (e.g. confidence/credible interval), ideally using structured tables or plots. | Table 1 |
| Results of syntheses | 20a | For each synthesis, briefly summarise the characteristics and risk of bias among contributing studies. | Table 1  Pg 5, line 2, line 8 |
|  | 20b | Present results of all statistical syntheses conducted. If meta-analysis was done, present for each the summary estimate and its precision (e.g. confidence/credible interval) and measures of statistical heterogeneity. If comparing groups, describe the direction of the effect. | Pg6-8 |
|  | 20c | Present results of all investigations of possible causes of heterogeneity among study results. | Pg 7, line 34  Pg 8, line 21 |
|  | 20d | Present results of all sensitivity analyses conducted to assess the robustness of the synthesized results. | NA |
| Reporting biases | 21 | Present assessments of risk of bias due to missing results (arising from reporting biases) for each synthesis assessed. | Pg 5, line 8 |
| Certainty of evidence | 22 | Present assessments of certainty (or confidence) in the body of evidence for each outcome assessed. | NA |
| **DISCUSSION** | | |  |
| Discussion | 23a | Provide a general interpretation of the results in the context of other evidence. | Pg 13, line 5 |
|  | 23b | Discuss any limitations of the evidence included in the review. | Pg 10, line 1 |
|  | 23c | Discuss any limitations of the review processes used. | Pg 10, line 2 |
|  | 23d | Discuss implications of the results for practice, policy, and future research. | Pg 9, line 48 |
| **OTHER INFORMATION** | | |  |
| Registration and protocol | 24a | Provide registration information for the review, including register name and registration number, or state that the review was not registered. | Pg 3, line 44 |
|  | 24b | Indicate where the review protocol can be accessed, or state that a protocol was not prepared. | Pg 3, line 45 |
|  | 24c | Describe and explain any amendments to information provided at registration or in the protocol. | Pg 5, line 6 |
| Support | 25 | Describe sources of financial or non-financial support for the review, and the role of the funders or sponsors in the review. | Pg 14, line 9 |
| Competing interests | 26 | Declare any competing interests of review authors. | Pg 14, line 7 |
| Availability of data, code and other materials | 27 | Report which of the following are publicly available and where they can be found: template data collection forms; data extracted from included studies; data used for all analyses; analytic code; any other materials used in the review. | NA |

*From:*  Page MJ, McKenzie JE, Bossuyt PM, Boutron I, Hoffmann TC, Mulrow CD, et al. The PRISMA 2020 statement: an updated guideline for reporting systematic reviews. BMJ 2021;372:n71. doi: 10.1136/bmj.n71

Supplementary Table 3. Meta-analyses of Observational Studies in Epidemiology (MOOSE) completed checklist

| **Item No** | **Recommendation** | **Reported on Page No** |
| --- | --- | --- |
| Reporting of background should include | | |
| 1 | Problem definition | 3 |
| 2 | Hypothesis statement | 3 |
| 3 | Description of study outcome(s) | 3 |
| 4 | Type of exposure or intervention used | 3 |
| 5 | Type of study designs used | 3 |
| 6 | Study population | 3 |
| Reporting of search strategy should include | | |
| 7 | Qualifications of searchers (eg, librarians and investigators) | 1 |
| 8 | Search strategy, including time period included in the synthesis and key words | 3 |
| 9 | Effort to include all available studies, including contact with authors | 4 |
| 10 | Databases and registries searched | 3 |
| 11 | Search software used, name and version, including special features used (eg, explosion) | 3 |
| 12 | Use of hand searching (eg, reference lists of obtained articles) | 3 |
| 13 | List of citations located and those excluded, including justification | Supp T2 |
| 14 | Method of addressing articles published in languages other than English | 3 |
| 15 | Method of handling abstracts and unpublished studies | 3 |
| 16 | Description of any contact with authors | 4 |
| Reporting of methods should include | | |
| 17 | Description of relevance or appropriateness of studies assembled for assessing the hypothesis to be tested | 4 |
| 18 | Rationale for the selection and coding of data (eg, sound clinical principles or convenience) | 4 |
| 19 | Documentation of how data were classified and coded (eg, multiple raters, blinding and interrater reliability) | 4 |
| 20 | Assessment of confounding (eg, comparability of cases and controls in studies where appropriate) | 4 |
| 21 | Assessment of study quality, including blinding of quality assessors, stratification or regression on possible predictors of study results | 4 |
| 22 | Assessment of heterogeneity | 4 |
| 23 | Description of statistical methods (eg, complete description of fixed or random effects models, justification of whether the chosen models account for predictors of study results, dose-response models, or cumulative meta-analysis) in sufficient detail to be replicated | 4 |
| 24 | Provision of appropriate tables and graphics | Table 1 |
| Reporting of results should include | | |
| 25 | Graphic summarizing individual study estimates and overall estimate | Table 1 |
| 26 | Table giving descriptive information for each study included | Table 1 |
| 27 | Results of sensitivity testing (eg, subgroup analysis) | 7,8 |
| 28 | Indication of statistical uncertainty of findings |  |
| Reporting of discussion should include | | |
| 29 | Quantitative assessment of bias (eg, publication bias) | 7, 8 |
| 30 | Justification for exclusion (eg, exclusion of non-English language citations) | NA |
| 31 | Assessment of quality of included studies | 4 |
| Reporting of conclusions should include | | |
| 32 | Consideration of alternative explanations for observed results | 13 |
| 33 | Generalization of the conclusions (ie, appropriate for the data presented and within the domain of the literature review) | 13 |
| 34 | Guidelines for future research | 13 |
| 35 | Disclosure of funding source | 14 |

*From*: Stroup DF, Berlin JA, Morton SC, et al, for the Meta-analysis Of Observational Studies in Epidemiology (MOOSE) Group. Meta-analysis of Observational Studies in Epidemiology. A Proposal for Reporting. *JAMA*. 2000;283(15):2008-2012. doi: 10.1001/jama.283.15.2008.

Supplementary Table 4. Records excluded at full-text screening with reasons (n=15)

| **Item number** | **Study reference** | **Justification for exclusion** |
| --- | --- | --- |
| 1 | Bakshi et al. Risk of adverse pregnancy outcomes after prior spontaneous abortion. Current Medical Research and Practice. 2015, 5(6):258-261 | Did not report confidence intervals |
| 2 | Challis et al. Gestational diabetes mellitus and fetal death in Mozambique: an incident case-reference study. Acta Obstetricia et Gyncologica Scandinavica. 2002, 81(6):560-563. | The exposure of pregnancy loss was mixed with stillbirth. |
| 3 | Cheung et al. Risk factors for gestational diabetes among Asian women. Diabetes Care. 2001, 24(5):955-956 | Did not distinguish between miscarriage and stillbirth in results |
| 4 | Cortes et al. Pregnancy loss is related to body mass index and prediabetes in early childhood: Findings from Add Health. PLoS One. 2022, 17(12):e0277320-e0277320. | The exposure of pregnancy loss was mixed with stillbirth. |
| 5 | Crane & Wahl. The role of maternal diabetes in repetitive spontaneous abortion. Fertility and Sterility. 1981, 36(4):447-479 | Did not report a suitable effect of association. |
| 6 | Dawson et al. A cohort study of reproductive risk factors, weight and weight change and the development of diabetes. Diabetes, Obesity & Metabolism. 2023, 5(4):244-250. | The exposure was mixed with the outcome of interest in index pregnancy. |
| 7 | Dorman et al. Temporal trends in spontaneous abortion associated with Type 1 diabetes. Diabetes Research and Clinical Practice. 1999, 43(1):41-47. | Did not report an effect of association. |
| 8 | Hiersch et al. The association between previous single first trimester abortion and pregnancy outcomes in nulliparous women. The Journal of Maternal-Fetal & Neonatal Medicine. 2016 29(9):1457-1461. | Outcomes were limited to mode of delivery. |
| 9 | Hung et al. Risk factors for gestational diabetes mellitus among women screened with the two-step and one-step methods: A before-and-after study. Taiwanese Journal of Obstetrics & Gynecology. 2018, 57(5):668-671. | Abortion was a confounding factor. |
| 10 | Kashanian et al. Pregnancy outcome following a previous spontaneous abortion (miscarriage). Human Reproduction 2020 35(2):275-282 | Did not report a suitable effect of association. |
| 11 | Keshavarz et al. Gestational diabetes in Iran: Incidence, risk factors and pregnancy outcomes. Gynecologic & Obstetric Investigation. 2006, 61(3):167-170. | The exposure group was unclear |
| 12 | Liang et al. Infertility, recurrent pregnancy loss and risk of stroke: Pooled analysis of individual patient data of 618 851 women. BMJ online. 2022, 377:e070603-e070603 | Did not report outcomes of interest. |
| 13 | Liu et al. Women with PCOS with a history of early pregnancy loss show a higher risk of gestational diabetes mellitus. International Journal of General Medicine. 2021, 14(1):6409-6416. | The exposure of early pregnancy loss was mixed with fertility. |
| 14 | Sikder et al. Risk factors for reported obstetric complications and near misses in rural northwest Bangladesh: Analysis from a prospective cohort study. BMC Pregnancy & Childbirth. 2014 14(1):347-347. | Did not distinguish between miscarriage and stillbirth in results. |
| 15 | Tai et al. A case-control study on risk factors for Type 1 diabetes in Taipei City. Diabetes Research and Clinical Practice. 1998, 42(3):197-203. | Outcome was in the child. |

Supplementary Table 5. Characteristics of the included studies (n=52)

| **First author (year of study), setting and study design** | **Study period** | **Sample size and study population** | **Exposure information** | **Outcome information** | **Unadjusted effect size measure (95% CI)** | **Adjusted effect size measure (95% CI)** |
| --- | --- | --- | --- | --- | --- | --- |
| Li (2023),^1^ China, Retrospective cohort | January 2000 and December 2019 | 111,124 women with singleton pregnancy (3,112 exposed and 108,012 not exposed) | RPL (≥2 SABs) | GDM  PE  GH | GDM:   - OR 1.12 (0.98-1.31)   PE:   - OR 1.21 (0.81-1.82)   GH:   - OR 1.28 (1.09-1.51) | GDM:   - OR 1.08 (0.92-1.41)   PE:   - OR 1.17 (0.77 – 1.93)   GH:   - OR 1.14 (0.98-1.28) |
| Sun (2023),^2^ China, Retrospective cohort | 2016 to 2020 | 75,773 women with live singleton births  (10,992 SAB; 13, 360 IA; 3,138 both SAB and IA; 48,283 controls) | SAB  IA  Both SAB + IA | GDM  PE  GH  HDP | GDM:   - SAB OR 1.54 (1.45-1.64) - IA OR 1.12 (1.01 – 1.23) - SAB + IA OR 1.52 (1.37-1.68)   PE:   - SAB OR 1.08 (0.95-1.24) - IA OR 0.81 (0.71-0.93) - SAB+IA OR 0.99 (0.70-1.15)   GH:   - SAB OR 1.36 (1.19-1.54) - IA OR 0.88 (0.76-1.01) - SAB+IA OR 1.12 (0.88-1.42)   HPD:   - SAB OR 1.22 (1.11-1.33) - IA OR 0.84 (0.76-1.01) - SAB+IA OR 1.00 (0.84-1.12) | GDM:   - SAB OR 1.25 (1.12 – 1.33) - IA OR 1.03 (0.97 – 1.1) - SAB + IA: OR 1.11 (1.00 – 1.26)   PE:   - SAB OR 0.91 (0.82-1.00) - IA OR 0.95 (0.83-1.09) - SAB+IA OR 0.85 (0.67-1.10)   GH:   - SAB OR 1.18 (1.03-1.35) - IA OR 0.97 (0.84 -1.13) - SAB+IA OR 1.10 (0.87-1.42)   HPD:   - SAB OR 1.02 (0.93-1.12) - IA OR 0.96 (0.87-1.06) - SAB+IA OR 0.96 (0.80-1.16) |
| Vaajala (2023)^3^,  Finland,  Retrospective cohort | 1^st^ January 2004 to 31^st^ December 2018 | 180,673 pregnant women (15,873 IA; 22,337 SAB; 3,594 both; 138,869 controls) | SAB  IA | GDM |  | SAB:   - All OR 1.14 (1.10-1.18) - 1 OR 1.13 (1.05-1.17) - 2 OR 1.18 (1.09-1.28) - 3+ OR 1.19 (1.04-1.36)   IA   - All OR 1.15 (1.10-1.20) - 1 OR 1.12 (1.07-1.17) - 2 OR 1.29 (1.17-1.42) - 3+ OR 1.48 (1.21-1.81) |
| Zhang (2023),^4^ China, Retrospective cohort | January 2014 and August 2021 | 108,792 first pregnancies (1,994 women with a history of recurrent (>2) miscarriages, 11,477 women with a history of one miscarriage, and 95,321 women with no history of miscarriage) | RPL (≥2 SABs) | GDM  PE | GDM:   - RR 1.53 (1.39-1.67)   PE:   - RR 1.35 (1.07-1.68) | GDM:   - RR 1.16 (1.06-1.26)   PE:   - RR 1.11 (0.88-1.38) |
| Askari (2022),^5^ Iran, Prospective cohort | March 2016 to February 2021 | 3,110 pregnant women (968 SAB; 335 RPL; 1922 controls) | SAB  RPL (≥2 SABs) | GDM |  | SAB:   - OR 1.05 (0.79 – 1.37)   RPL:   - OR 1.41 (0.98- 2.03) |
| Mohamedain (2022)^6^, Sudan, Case-control | February - December 2020 | 360 pregnant women (180 cases and 180 controls) | SAB | PE | OR 0.47 (0.29-0.74) | OR 0.44 (0.23-0.73) |
| Okoth (2022)^7^, UK, Retrospective cohort | 1^st^ January 1995 to 15^th^ May 2016 | 416,374 women aged 16-50 years (86,509 exposed and 329,865 unexposed) | SAB | Diabetes  Hypertension | Diabetes:   - IRR 1.10 (1.05–1.16)   Hypertension:   - IRR 1.10 (1.05–1.16) | Diabetes:   - IRR 1.07 (1.02–1.12)   Hypertension:   - IRR 1.07 (1.02–1.12) |
| Taye (2022)^8^, Ethiopia,  Case-control | 25^th^ January 2020 through 25^th^ April 2020. | 320 pregnant women (80 cases and 240 controls) | SAB | GDM | OR 2.08 (1.23-3.50) | OR 2.40 (1.33-4.31) |
| Zhao (2022)^9^, China, Retrospective cohort | January 2014 – December 2019 | 102,259 pregnant women  (SAB 14,579; IA 17,935; Both 4,017 Controls 65,998) | SAB  IA  Both SAB and IA | GDM | SAB:   - RR 1.56 (1.43-1.63)   IA:   - RR 1.18; (1.12 to 1.24)   Both SAB and IA:   - RR 1.56 (1.45-1.73) | SAB   - RR 1.25 (1.18-1.31)   IA:   - RR 1.04 (0.98-1.10)   Both SAB and IA:   - RR 1.15 (1.05-1.27) |
| Ausbeck (2021)^10^,  US,  Retrospective cohort | 1^st^ January 2008 to 31^st^ December 2017 | 17,670 pregnant women (235 exposed and 17,435 unexposed) | RPL (≥2 SAB) | GDM  PE |  | GDM:   - OR 1.69 (1.10 – 2.59)   PE:   - OR 0.87 (0.56- 1.36) |
| Huo (2021)^11^, China,  Cross-sectional | 2011-2012 | 131,174 women aged ≥ 40 years (30,020 cases and 101,154 controls) | SAB | Type 2 diabetes | - 1 OR 0.93 (0.90-0.96) - 2 OR 0.94 (0.91-0.98) - ≥ 3 OR 0.90 (0.86-0.94) | - 1 OR 1.04 (0.98-1.09) - 2 OR 1.09 (1.04-1.14)   ≥3 OR 1.11 (1.04-1.18) |
| Roepke (2021)^12^, Sweden, Retrospective cohort | 2003-2012 | 62,381 pregnancies (4971 exposed and 57410 non-exposed) | RPL (≥2 SAB) | PE | Any PE:   - OR 1.97 (1.70-2.28)   Mild/moderate PE   - OR 1.87 (1.57 - 2.23)   Severe PE   - OR 2.22 (1.70 - 2.90) | Any PE:   - OR 1.45 (1.24-1.69)   Mild/moderate PE   - OR 1.39 (1.17 to 1.68)   Severe PE   - OR 1.57 (1.20 - 2.07) |
| Stitterich (2021)^13^, Sierra Leone, Case-control | November 2018 – February 2019 | 672 pregnant women (214 cases and 458 controls) | IA | PE | IA   - OR 0.50 (0.24 – 1.05) |  |
| Ali (2020)^14^, United Arab Emirates, Prospective cohort | May 2017 – April 2019 | 1,737 pregnancy women (234 exposed 1503 unexposed) | RPL (≥2 SAB) | GDM  PE | GDM:   - OR 0.90 (0.65-1.27)   PE:   - OR 2.74 (1.12-6.67) | GDM:   - OR 0.69 (0.47 – 1.02)   PE:   - OR 2.81 (0.95-8.27) |
| Christians (2020)^15^, US, Cohort | 1959-1965 | 5,449 women (195 cases; 3,670 controls) | SAB | PE | OR 1.4 (0.7-2.8) | OR 1.1 (0.5-2.4) |
| Egerup (2020)^16^, Denmark, Nested control-case | 1997-2017 | 272,514 women born between 1957 and 1997 (24,774 women exposed and 247,740 unexposed) | Pregnancy loss (undefined) | Type 2 diabetes | - 1 OR 1.42 (1.28 – 1.58) - 2 OR 1.74 (1.41 – 2.16) - 3+ OR 2.84 (2.09 – 3.87) | - 1 OR 1.40 (1.25 – 1.58) - 2 OR 1.71 (1.35 – 2.17) - 3+ OR 2.79 (1.98 – 3.94) |
| Peters (2020)^17^,  China,  Cohort | 2004 – 2016 | 273,383 women aged 30-79 years (165,508 exposed; unexposed 168,110) | SAB  IA | Type 2 diabetes | SAB   - 1 HR 1.02 (0.93-1.11) - 2 + HR 1.05 (0.89 – 1.25)   IA   - 1 HR 1.05 (1.01-1.09) - 2 + HR 1.10 (1.10 – 1.15)   Both SAB and IA   - 1 HR 1.04 (1.00-1.08) - 2 HR 1.08 (1.03 – 1.14) - 3 HR 1.17 (1.08 – 1.27) - 4+ HR 1.16 (1.04 – 1.30) | SAB   - 1 HR 1.02 (0.94-1.11) - 2 + HR 1.06 (0.90 – 1.26)   IA   - 1 HR 1.06 (1.02-1.10) - 2+ HR 1.09 (1.04 – 1.14)   Both SAB and IA   - 1 HR 1.05 (1.01-1.09) - 2 HR 1.08 (1.02 – 1.14) - 3 HR 1.16 (1.07 – 1.26) - 4+ HR 1.1 (1.01 – 1.26) |
| Sembiring (2020)^18^, Indonesia, Cross-sectional | Not clear | 100 pregnant women | SAB | HDP | OR 1.61 (0.53-4.92) |  |
| Su (2020)^19^, China, Prospective cohort | June 2013 – November 2014 | 5191 pregnant women (1349 exposed and 3789 not exposed) | IA | PE  GH  HDP | PE:   - OR 0.67 (0.42-1.06)   GH:   - OR 0.88 (0.59-1.30)   HDP:   - OR 0.78 (0.57-1.05) | PE:   - OR 0.61 (0.38-0.97)   GH:   - OR 0.73 (0.49-1.10)   HDP:   - OR 0.67 (0.49-0.91) |
| Ticconi (2020)^20^, Italy,  Prospective cohort | 1^st^ January 2017 to 31^st^ January 2020 | 1,092 pregnancies (431 exposed and 661 non exposed) | RPL (≥2 SAB) | GDM  PE |  | GDM:   - 2 OR 2.39 (1.35-4.23) - 3 OR 2.14 (1.03 – 4.40) - >3 OR 1.93 (0.97 – 3.83)   PE:   - 2 OR 2.76 (1.63-4.67) - 3 OR 1.93 (0.97 – 3.83) - >3 OR 1.15 (0.47 – 2.21) |
| Unnikrishnan (2020)^21^, India, Case-control | January 2018 and December 2018 | 262 pregnant women (131 cases and 131 controls) | SAB | GDM | OR 1.26 (0.69 – 2.29) |  |
| Horn (2019)^22^, US, Prospective cohort | 2009 | 60,651 female registered nurses (6,587 SAB; 7,937 IA; 46,127 controls) | Early SAB (< 12 weeks)  Late SAB (12-1 weeks)  IA | Type 2 diabetes  Hypertension |  | Type 2 diabetes:  Early SAB   - 1.11 (1.01 – 1.22) - 1 HR 1.11 (1.00-1.22) - 2 HR 1.08 (0.88-1.32) - 3+ HR 1.43 (1.06-1.92)   Late SAB:   - Ever HR 1.17 (1.02- 1.33) - 1 HR 1.18 (1.31-1.35) - 2+ HR 1.04 (0.72-1.51)   IA:   - Ever HR 0.92 (0.84-1.02) - 1 HR 0.97 (0.87-1.08) - 2 HR 0.76 (0.61-0.95) - 3+ HR 0.92 (0.63-1.35)   Hypertension:  Early SAB   - Ever HR 1.05 (1.00 – 1.09) - 1 HR 1.04 (1.00-1.09) - 2 HR 1.04 (0.95-1.14) - 3+ HR 1.19 (1.03-1.38)   Late SAB:   - Ever HR 1.12 (1.05- 1.18) - 1 HR 1.11 (1.05-1.19) - 2+ HR 1.13 (0.95-1.35)   IA:   - Ever HR 0.88 (0.84-0.92) - 1 HR 0.91 (0.87-0.96) - 2 HR 0.79 (0.72-0.86) - 3+ HR 0.75 (0.63-0.90) |
| Cozzolino (2019)^23^, Italy, Retrospective cohort | November 2014 – December 2015 | 118 (53 exposed in and 65 in the control group) | RPL (≥2 SABs) | PE  GH | PE: OR 1.23 (0.08 - 20.16)  GH: OR 1.24 (0.17–9.08) |  |
| Feleke (2019)^24^, Ethiopia, Unmatched case-control | January 2016 to June 2016 | 2257 pregnant women (567 cases; 1690 controls) | SAB | GDM | OR 3.76 (2.46–5.75) | OR 5.05 (2.65–9.63) |
| Lao (2018)^25^, Hong Kong, Retrospective cohort | 1997 – 2015 | 60,335 women (36637 unexposed and 23698 exposed (1 abortion 15167, 2 or more 8531)) | Abortion (mixed SAB and IA) | PE  GH  HDP |  | PE:   - RR 0.86 (0.75–0.99)   GH:   - RR 0.66 (0.56–0.77)   HDP:   - RR 0.76 (0.68–0.85) |
| Liu (2018)^26^, China,  Cross-sectional | Not clear | 19,539 middle aged and older Chinese women (3664 cases 15875 controls) | SAB | Diabetes | - 1 OR 0.93 (0.76 – 1.15) - 2 OR 1.33 (0.89- 2.00) - 3+ OR 2.24 (1.25 – 4.02) | - 1 OR 0.84 (0.67 – 1.55) - 2 OR 1.26 (0.82 – 1.96) - 3+ OR 2.01 (1.07 – 3.78) |
| Yang (2018)^27^, China, Cross-sectional | August 2013 – August 2015 | 6,252 Postmenopausal women aged 41–93 years (1530 pre-hypertension, 3496 hypertension) | SAB  IA | Hypertension |  | Hypertension:  SAB   - OR 0.97 (0.77 - 1.22)   IA   - OR 1.19 (1.02 - 1.39) |
| Liu (2017)^28^, China,  Retrospective case-control | 2014-2015 | 600 pregnancy women (300 cases with GDM and 300 controls) | SAB  IA | GDM | SAB:  OR 5.44 (2.44-12.10)  IA:  OR 15.54 (5.82-41.49) |  |
| Sepidarkish (2017)^29^, Iran, Cross-sectional | 6^th^ July – 21^st^ July 2015 | 5,170 pregnancy women (252 cases 4918 controls) | SAB | PE | OR 1.34 (1.10–1.63) | OR 1.28 (1.03–1.59) |
| Su (2017)^30^, China, Case-control | 2014 – 2015 | 600 (300 cases and 300 controls) | SAB | PE | OR 1.3 (0.7-2.1) |  |
| AlKasseh (2014)^31^, Gaza,  Retrospective cohort | March – June 2011 | 378 pregnant refugee women (189 cases and 189 controls) | SAB | GDM | - 1 OR 4.02 (2.33– 6.91) - 1 + OR 5.98 (3.16– 11.30) | - 1 OR 3.11 (1.55– 6.21) - 1+ OR 4.93 (2.20– 11.04) |
| Gunnarsdottir (2014)^32^, Sweden, Prospective cohort | 1995 – 2009 | 619,587 primiparous women (1SAB 68,185; 2 SAB 11,410; ≥3 SAB 3,823, unexposed 536,169) | SAB | PE |  | Pre-eclampsia:   - 1 OR 0.98 (0.94 - 1.02) - 2 OR 0.94 (0.85 - 1.04) - ≥3 OR 1.23 (1.06 - 1.42) |
| Makhlouf (2014)^33^, US, Prospective cohort | July 2003 – February 2008 | 9,738 pregnant women (7681 primigravid women (unexposed) 2057 (exposed: 1240 (1 or more SAB) 817 (1 or more induced)) | SAB | PE |  | - 1 SAB OR 1.0 (0.7–1.2) - 2 SABs OR 0.6 (0.3–1.2) |
| Parker (2014)^34^, Finland, Cross-sectional | 1996 – 2010 | 63,250 pregnant women (12,650 preeclampsia cases and 50,600 controls) | IA | PE | - Ever IA OR 0.9 (0.8 - 0.9) - 1 IA OR 0.9 (0.9 - 1.0) - 2 IA OR 0.8 (0.7 - 0.9) - ≥3 IAs OR 0.7 (0.5 – 1.0) |  |
| Ranthe (2013)^35^, Denmark, Retrospective cohort | 1977 – 2008 | 1,031,279 ≥12 years with at least 1 pregnancy ending in a live singleton birth, (188,663 had ≥1 SABs (exposed) 842 616 women no loss (unexposed)) | SAB | Hypertension |  | - 1 SAB IRR 1.15 (0.99–1.34) - 2 SAB IRR 1.12 (0.80–1.56) - 3 SAB IRR 1.78 (1.00–3.14) - ≥4 SAB IRR 3.78 (2.08–6.85) |
| Kharazmi (2012)^36^, Germany,  Prospective cohort | 1994-1998 | 11,518 women aged 35-65 years with reproductive history (2,876 had at least one SAB, 2,053 had at least one IA) | SAB  IA | Diabetes | SAB   - 1 HR 1.37 (1.07 - 1.77) - 1- 2 HR 1.34 (1.03 1.74 - 2+ HR 1.91 (0.94 - 3.87)   IA:   - HR 0.70 (0.46 - 1.05) - 1-2 HR 0.70 (0.46 - 1.07) - 2+ HR 0.65 (0.16 – 2.59) | SAB   - 1 OR 1.06 (0.90 - 1.25) - 1-2 OR 1.00 (0.84 1.19) - 2+ OR 1.90 (1.25 – 2.90)   IA:   - OR 0.71 (0.56 – 0.90) - 1-2 OR 0.70 (0.55 – 0.90) - 2+ OR 1.00 (0.36 – 2.82) |
| Weintraub (2011)^37^, Israel, Retrospective cohort | 1998-2008 | 35,125 pregnant women (5777 exposed and 29348 unexposed) | SAB | GDM  PE  GH | GDM:   - OR 1.45 (1.29–1.64)   PE:   - OR 2.38 (2.16–2.67)   GH:   - OR 1.42 (1.12–1.80) | PE:   - OR 1.63 (1.22–2.18)   CH:   - OR 1.41 (1.07–1.85) |
| Bhat (2010)^38^, India, Case-control | August 2007 – June 2008 | 600 pregnant women (300 cases and 300 controls) | SAB  IA | GDM | Pregnancy loss:   - OR 2.4 (1.3-4.3)   IA:   - OR 0.9 (0.6-1.3) |  |
| Olayemi (2010)^39^, Nigeria, Prospective cohort | Not clear | 1,850 nulliparous women (605 cases; 1,245 controls) | SAB | GH |  | OR 1.25 (0.83–1.88) |
| Trogstad (2009)^40, 41^*reported twice for SAB and Pre-eclampsia, Norway, Prospective cohort | 1999 – 2005 | 20,846 singleton pregnancies (SAB 1,112; unexposed 20,846) | SAB | PE | - 1 SAB OR 0.96 (0.80–1.16) - 2 SAB OR 0.81 (0.52–1.27) - 3+ SAB OR 1.62 (0.87–3.01) | - 1 SAB OR 0.95 (0.78–1.14) - 2 SAB OR 0.78 (0.50–1.22) - 3+ SAB OR 1.51 (0.80–2.83) |
| Yang (2009)^42^, China, Prospective cohort | 1^st^ April 2006 - 30^th^ September 30 2006 | 16,286 pregnant women (708 exposed and 15,578 unexposed) | SAB | GDM | OR 1.49 (1.13–1.99) | OR 1.46 (1.12–1.91) |
| Bhattacharya (2008)^43^, Scotland, Retrospective cohort | 1986 - 2000 | 33,228 women (1,561 exposed; 31,667 unexposed) | SAB | PE | OR 3.2 (2.4–4.3) | OR 3.3 (2.6–4.6) |
| Trogstad (2009)^40, 41^*reported twice for SAB and Pre-eclampsia, Norway, Prospective cohort | 1999 – 2005 | 20,846 singleton pregnancies | IA | PE | - 1 IA OR 0.84 (0.69–1.02) - 2+ IA OR 0.36 (0.18–0.73) | - 1 IA OR 0.87 (0.71–1.06) - 2+ IA OR 0.39 (0.19–0.80) |
| Sheiner (2005)^44^, Israel, Retrospective cohort | 1998-2002 | 154,294 singleton births (7503 exposed and 146791 unexposed) | RPL (≥2 SABs) | PE  GH | Mild PE:   - OR 1.0 (0.9 – 1.2)   Severe PE:   - OR 1.5 (1.3-1.8)   GH:   - OR 2.2 (2.0 – 2.5) |  |
| Vanek (2004)^45^, Israel, Cross-sectional | 1998 – 1999 | 114,963 births (1807 with chronic hypertension) | Recurrent abortion (undefined) | GH | OR 1.5 (1.3 – 1.8) |  |
| Saftlas (2003)^46^, US, Prospective cohort | 1992 – 1999 | 4,895 women (247 with 2 or more abortions) | SAB | PE  GH | PE (same father):   - OR 0.50 (0.28 – 0.89)   PE (different father):   - OR 0.98 (0.69 – 1.39)   GH (same father):   - OR 0.85 (0.58 – 1.11)   GH (different father):   - OR 0.96 (0.75 – 1.23) | PE (same father):   - OR 0.55 (0.31 – 0.97)   PE (different father):   - OR 1.03 (0.72 – 1.47)   GH (same father):   - OR 0.83 (0.59 – 1.15)   GH (different father):   - OR 0.99 (0.77 – 1.27) |
| Xiong (2002)^47^, Canada, Retrospective cohort | 1^st^ January 1993- 21^st^ December 1999 | 51,430 pregnant women (39,818 unexposed, 11,612 exposed (8,888 one abortion; 1,951 two abortions; 773 three abortions) | SAB | PE | - Ever OR 0.91 (0.80 – 1.03) - 1 OR 0.85 (0.74 – 0.99) - 2 0.89 (0.67 – 1.19) - ≥3 OR 1.56 (1.10 – 2.22) | - Ever OR 0.89 (0.78 – 1.01) - 1 OR 0.84 (0.72 – 0.97) - 2 OR 0.89 (0.67 – 1.19) - ≥3 OR 1.42 (0.99 – 2.03) |
| Eras (2000)^48^, US, Cohort | 5^th^ April 1988 – 31^st^ December 1991 | 2739 singleton births (44 exposed to pre-eclampsia; 172 exposed to gestational hypertension) | SAB  IA | PE  GH | PE:   - 1 SAB OR 0.23 (0.00 – 1.43) - 2 SAB OR 0.30 (0.07 – 1.86) - 1 IA OR 0.42 (0.00 – 1.38) - 2 IA OR 0.30 (0.00 – 1.86) - SAB & IA OR 0.87 (0.20 – 3.62)   GH:   - 1 SAB OR 1.23 (0.61 – 2.31) - 2 SAB OR 2.24 (0.53 – 7.19) - 1 IA OR 1.01 (0.55 – 1.75) - 2 IA OR 0.11 (0.00 – 0.67) - SAB & IA OR 0.33 (0.04 – 1.31) |  |
| Sibai (1997)^49^, US, Prospective cohort | Not stated | 4,314 pregnancies survived past 20 gestational weeks (3,242 unexposed and 1,072 exposed (1 abortion 825, 2 or more 247)) | SAB | PE | - 1 SAB OR 0.81 (0.60 – 1.10) - ≥2 SAB OR 0.70 (0.40 – 1.21) | - 1 SAB OR 0.86 (0.63 – 1.19) - ≥2 SAB OR 0.73 (0.41 – 1.31) |
| Parazzini (1996)^50^, Italy, Prospective cohort | Not stated | 756 women (SAB: exposed 142 unexposed 614; Induced: exposed 50 and unexposed 706)) | SAB  IA | GH |  | SAB   - OR 1.0 (0.6 – 1.7)   IA   - OR 1.2 (0.6 – 2.5) |
| Abi-Said (1995)^51^, US, Case-control | 1997 – 1992 | 330 (66 cases and 264 matched controls) | SAB | PE | OR 0.23 (0.09 – 0.59) |  |
| Eskenazi (1991)^52^, US, Case-control | 1984 – 1985 | 271 (139 cases and 132 controls) | SAB  IA | PE | SAB   - OR 0.48 (0.24 – 0.95)   IA   - OR 2.16 (1.18 – 3.96) | SAB   - OR 0.31 (0.13 – 0.74)   IA   - OR 10.8 (1.2 – 29.1) |

SAB spontaneous abortion; IA induced abortion; RPL repeated pregnancy loss; GDM gestational diabetes mellitus; PE pre-eclampsia; GH gestational hypertension; HPD hypertensive disorders of pregnancy; OR odds ratio; RR relative risk; HR hazard ratio; IRR incidence rate ratio

Supplementary Table 6: Risk of bias assessment of the included studies based on the Risk of Bias in Non-randomized Studies of Exposure (ROBINS-E) tool

| **First author (year)** | **D1** | **D2** | **D3** | **D4** | **D5** | **D6** | **D7** | **Overall** |
| --- | --- | --- | --- | --- | --- | --- | --- | --- |
| Li (2023) | High risk | Some concerns | Some concerns | Low risk | Low risk | Low risk | Low risk | High risk |
| Sun (2023) | Some concerns | Low risk | Low risk | Low risk | Low risk | Low risk | Low risk | Some concerns |
| Vaajala (2023) | Low risk | Low risk | Some concerns | Low risk | Low risk | Low risk | Some concerns | Some concerns |
| Zhang (2023) | Low risk | Low risk | Low risk | Low risk | Low risk | Low risk | Low risk | Low risk |
| Askari (2022) | Low risk | Low risk | High risk | Low risk | Low risk | Low risk | Some concerns | High risk |
| Mohamedain (2022) | Low risk | Low risk | Some concerns | Low risk | Low risk | Low risk | Some concerns | Some concerns |
| Okoth (2022) | Some concerns | Low risk | Low risk | Low risk | Low risk | Low risk | Low risk | Some concerns |
| Taye (2022) | High risk | Low risk | High risk | Low risk | Low risk | Low risk | Some concerns | High risk |
| Zhao (2022) | Low risk | Low risk | Some concerns | Low risk | Low risk | Low risk | Low risk | Some concerns |
| Ausbeck (2021) | Low risk | Low risk | Low risk | Low risk | Low risk | Low risk | Low risk | Low risk |
| Huo (2021) | Low risk | Low risk | Low risk | Low risk | Some concerns | Low risk | Low risk | Some concerns |
| Roepke (2021) | Low risk | Low risk | Low risk | Low risk | Low risk | Low risk | Low risk | Low risk |
| Stitterich (2021) | Very high risk | Very high risk | Very high risk | Very high risk | Very high risk | Very high risk | Very high risk | Very high risk |
| Ali (2020) | High risk | Low risk | Low risk | Low risk | Low risk | Low risk | Low risk | High risk |
| Christians (2020) | Some concerns | Low risk | Low risk | Low risk | Some concerns | Low risk | Low risk | Some concerns |
| Egerup (2021) | Very high risk | Low risk | Low risk | Low risk | Low risk | Low risk | Low risk | Very high risk |
| Peters (2020) | Low risk | Low risk | Some concerns | Low risk | Some concerns | Low risk | Low risk | Some concerns |
| Sembiring (2019) | Very high risk | Very high risk | Very high risk | Very high risk | Very high risk | Very high risk | Very high risk | Very high risk |
| Su (2020) | Low risk | Low risk | Low risk | Low risk | Low risk | Low risk | Low risk | Low risk |
| Ticconi (2020) | High risk | Low risk | Low risk | Low risk | Low risk | Low risk | Low risk | High risk |
| Unnikrishnan (2020) | Very high risk | Very high risk | Very high risk | Very high risk | Very high risk | Very high risk | Very high risk | Very high risk |
| Horn (2018) | Low risk | Low risk | Low risk | Low risk | Low risk | Low risk | Low risk | Low risk |
| Cozzolino (2019) | Very high risk | Very high risk | Very high risk | Very high risk | Very high risk | Very high risk | Very high risk | Very high risk |
| Feleke (2018) | Low risk | Low risk | High risk | Low risk | Low risk | Some concerns | Low risk | High risk |
| Lao (2018) | Some concerns | Low risk | High risk | Low risk | Low risk | Low risk | Low risk | High risk |
| Liu (2018) | Low risk | Low risk | Low risk | Low risk | Low risk | Low risk | Low risk | Low risk |
| Yang (2018) | Low risk | Low risk | Low risk | Low risk | Some concerns | Low risk | Low risk | Some concerns |
| Liu (2017) | Very high risk | Very high risk | Very high risk | Very high risk | Very high risk | Very high risk | Very high risk | Very high risk |
| Sepidarkish (2016) | Some concerns | Low risk | Low risk | Low risk | Low risk | Low risk | Low risk | Some concerns |
| Su (2017) | Very high risk | Very high risk | Very high risk | Very high risk | Very high risk | Very high risk | Very high risk | Very high risk |
| AlKasseh (2013) | Low risk | Low risk | High risk | Low risk | Low risk | Low risk | Low risk | High risk |
| Gunnarsdottir (2014) | Low risk | Low risk | Low risk | Low risk | Low risk | Low risk | Low risk | Low risk |
| Makhlouf (2014) | Low risk | Low risk | Low risk | Low risk | Low risk | Low risk | Low risk | Low risk |
| Parker (2015) | Very high risk | Very high risk | Very high risk | Very high risk | Very high risk | Very high risk | Very high risk | Very high risk |
| Ranthe (2013) | High risk | Low risk | Low risk | Low risk | Low risk | Low risk | Low risk | High risk |
| Kharazmi (2012) | Low risk | Low risk | Low risk | Low risk | Low risk | Low risk | Low risk | Low risk |
| Weintraub (2011) | High risk | Some concerns | Some concerns | Low risk | Low risk | Low risk | Low risk | High risk |
| Bhat (2010) | Very high risk | Very high risk | Very high risk | Very high risk | Very high risk | Very high risk | Very high risk | Very high risk |
| Olayemi (2010) | Low risk | Low risk | Low risk | Low risk | Low risk | Low risk | Low risk | Low risk |
| Trogstad (2009) | Low risk | Low risk | Low risk | Low risk | Low risk | Low risk | Low risk | Low risk |
| Yang (2009) | Low risk | Low risk | Low risk | Low risk | Low risk | Low risk | Low risk | Low risk |
| Bhattacharya (2008) | Low risk | Low risk | Low risk | Low risk | Low risk | Low risk | Low risk | Low risk |
| Trogstad (2008) | Low risk | Low risk | Low risk | Low risk | Low risk | Low risk | Low risk | Low risk |
| Sheiner (2005) | Very high risk | Very high risk | Very high risk | Very high risk | Very high risk | Very high risk | Very high risk | Very high risk |
| Vanek (2004) | Very high risk | Very high risk | Very high risk | Very high risk | Very high risk | Very high risk | Very high risk | Very high risk |
| Saftlas (2003) | Low risk | Low risk | Low risk | Low risk | Low risk | Low risk | Low risk | Low risk |
| Xiong (2002) | Low risk | Low risk | Low risk | Low risk | Low risk | Low risk | Low risk | Low risk |
| Eras (2000) | Very high risk | Very high risk | Very high risk | Very high risk | Very high risk | Very high risk | Very high risk | Very high risk |
| Sibai (1997) | Low risk | Some concerns | Low risk | Low risk | Low risk | Low risk | Some concerns | Some concerns |
| Parazzini (1996) | Low risk | Some concerns | High risk | Low risk | Some concerns | Low risk | Some concerns | Some concerns |
| Abi-Said (1995) | Very high risk | Very high risk | Very high risk | Very high risk | Very high risk | Very high risk | Very high risk | Very high risk |
| Eskenazi (1991) | Some concerns | Low risk | Some concerns | Low risk | Some concerns | Low risk | Some concerns | Some concerns |

D1 Bias due to confounding; D2 Bias due to selection of participants; D3 Bias due to misclassification of exposures; D4 bias due to post-exposure interventions; D5 Bias due to missing data; D6 Bias in the measurement of the outcomes; D7 Bias in selection of the reported result.

Supplementary Table 7. Confounders included in the analysis of the association between early pregnancy loss and the development of a diabetic or a hypertensive disorder

| **First Author (Year)** | **Maternal age** | **Parity** | **BMI** | **Smoking** | **Drinking** | **Education** | **Ethnicity** | **SES** | **ART** | **Gravidity** | **Reproductive history** | **Comorbidities** | **History of disorders** | **Medical profile** | **Nutritional status** | **Physical activity** | **Birth outcomes** |
| --- | --- | --- | --- | --- | --- | --- | --- | --- | --- | --- | --- | --- | --- | --- | --- | --- | --- |
| Sun (2023) | ● | ● | ● |  |  |  |  |  |  |  | ● |  |  |  |  |  | ● |
| Vaajala (2023) | ● | ● | ● | ● |  |  |  |  | ● | ● |  |  |  |  |  |  |  |
| Zhang (2023) | ● | ● | ● |  |  | ● |  | ● | ● |  |  | ● | ● |  |  |  |  |
| Askari (2023) | ● | ● | ● | ● |  | ● |  | ● |  |  | ● | ● | ● | ● |  |  | ● |
| Mohamedin (2022) | ● | ● | ● |  |  | ● |  | ● |  | ● |  | ● | ● | ● |  |  | ● |
| Okoth (2022) | ● |  | ● | ● |  |  |  | ● | ● |  |  | ● |  | ● |  |  |  |
| Taye (2022) |  |  |  |  |  |  |  |  |  | ● | ● |  |  | ● | ● |  |  |
| Zhao (2022) | ● | ● | ● |  |  |  |  |  | ● |  | ● |  |  | ● |  |  |  |
| Ausbeck (2021) | ● | ● | ● |  |  |  | ● |  |  |  |  | ● |  |  |  |  |  |
| Huo (2021) | ● | ● | ● | ● | ● | ● |  |  |  |  | ● |  | ● | ● |  | ● |  |
| Roepke (2021) | ● | ● | ● | ● |  |  | ● |  | ● |  |  | ● |  |  |  |  |  |
| Ali (2020) | ● |  | ● |  |  |  |  |  |  | ● |  |  |  |  |  |  | ● |
| Christians (2020) |  |  | ● | ● |  |  | ● |  |  |  | ● |  |  |  |  |  | ● |
| Egerup (2020) |  |  | ● |  |  |  |  |  |  |  |  |  |  |  |  |  |  |
| Peters (2020) |  | ● | ● | ● | ● | ● |  |  |  | ● |  | ● |  |  |  |  |  |
| Su (2020) | ● |  | ● |  |  | ● |  |  |  |  |  | ● | ● |  |  |  |  |
| Ticconi (2020) | ● |  | ● |  |  |  |  |  |  |  |  |  |  |  |  |  |  |
| Horn (2019) | ● |  | ● | ● | ● | ● | ● |  |  |  |  |  |  |  | ● | ● |  |
| Feleke (2018) |  | ● | ● |  |  | ● |  |  |  |  | ● | ● | ● |  | ● | ● |  |
| Lao (2018) | ● |  | ● |  |  |  |  |  |  |  |  | ● | ● | ● |  |  |  |
| Liu (2018) | ● | ● | ● | ● | ● | ● |  |  |  |  | ● | ● |  |  |  | ● |  |
| Yang (2018) | ● |  | ● | ● | ● | ● |  |  |  |  |  |  |  | ● |  | ● |  |
| Sepidarkish (2017) | ● | ● | ● |  |  |  |  |  |  |  | ● |  |  |  |  |  |  |
| Alkasseh (2014) | ● |  |  |  |  |  |  |  |  | ● |  | ● |  |  |  |  |  |
| Gunnarsdottir (2014) | ● |  | ● | ● |  | ● | ● |  | ● |  | ● | ● |  |  |  |  | ● |
| Makhlouf (2014) | ● |  | ● | ● |  | ● | ● |  |  |  |  |  |  |  |  |  |  |
| Ranthe (2013) | ● |  |  |  |  |  |  |  |  | ● |  |  |  |  |  |  | ● |
| Kharazmi (2012) | ● |  | ● | ● | ● | ● |  |  |  |  | ● | ● |  | ● |  | ● |  |
| Weintraub (2011) | ● |  |  |  |  |  |  |  |  |  |  |  |  |  |  |  |  |
| Olayemi (2010) | ● |  | ● |  |  |  |  |  |  |  |  |  |  | ● |  |  |  |
| Trogstad (2009) | ● |  | ● | ● |  | ● |  |  |  |  | ● |  |  |  |  |  |  |
| Yang (2009) | ● |  | ● |  |  | ● |  |  |  |  |  |  | ● |  |  |  |  |
| Bhattacharya (2008) | ● |  | ● | ● |  |  |  | ● |  |  | ● |  |  |  |  |  |  |
| Trogstad (2008) | ● |  | ● | ● |  | ● |  |  | ● |  |  |  |  |  |  |  |  |
| Saftlas (2003) |  |  | ● | ● |  |  |  |  |  |  |  |  |  |  |  |  |  |
| Xiong (2002) | ● | ● |  | ● |  |  |  |  |  |  | ● | ● |  |  |  |  |  |
| Sibai (1997) | ● |  | ● | ● |  |  |  |  |  |  |  | ● |  |  |  |  |  |
| Parazzini (1996) | ● | ● |  |  |  |  |  |  |  |  |  |  |  |  |  |  |  |
| Eskenazi (1991) |  | ● | ● | ● |  |  |  |  |  |  | ● |  |  |  |  |  |  |

**
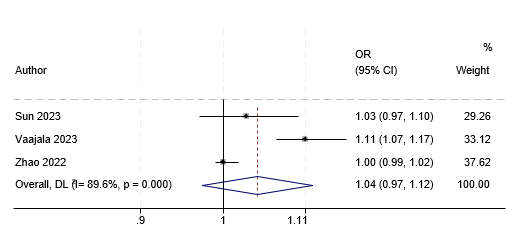
**

**OR: Odds ratio. CI: Confidence interval, p: P-value**

Supplementary Figure 1. Meta-analysis of the association between a prior induced abortion and the risk of developing gestational diabetes mellitus in a subsequent pregnancy.


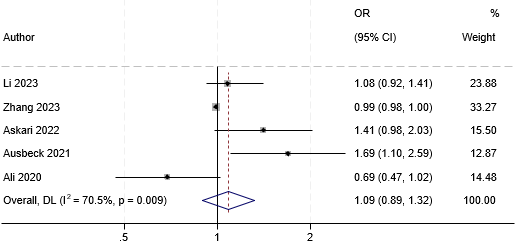


**OR: Odds ratio. CI: Confidence interval, p: P-value**

Supplementary Figure 2. Meta-analysis of the association between a recurrent pregnancy loss and the risk of developing gestational diabetes mellitus in a subsequent pregnancy**.**


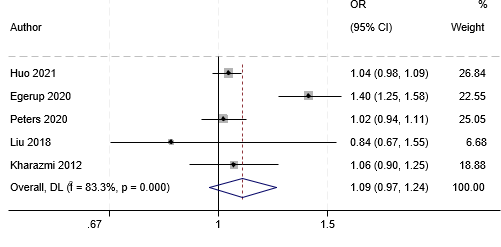


**OR: Odds ratio. CI: Confidence interval, p: P-value**

Supplementary Figure 3. Meta-analysis of the association between a spontaneous abortion and the risk of developing non-pregnancy related diabetes.


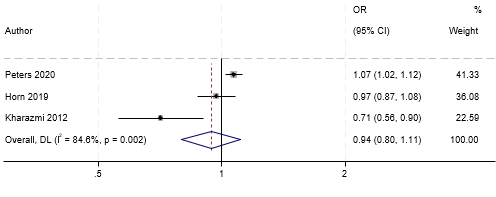


**OR: Odds ratio. CI: Confidence interval, p: P-value**

Supplementary Figure 4. Meta-analysis of the association between an induced abortion and the risk of developing non-pregnancy related diabetes.


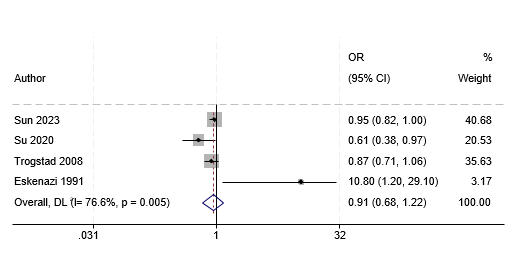


**OR: Odds ratio. CI: Confidence interval, p: P-value**

Supplementary Figure 5. Meta-analysis of the association between an induced abortion and the risk of developing pre-eclampsia in a subsequent pregnancy.


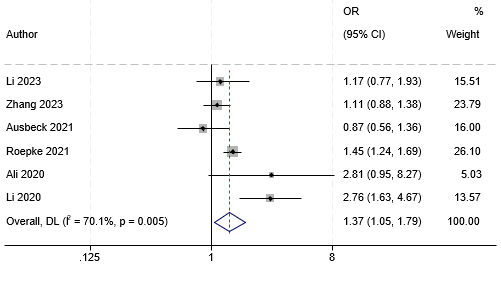


**OR: Odds ratio. CI: Confidence interval, p: P-value**

Supplementary Figure 6. Meta-analysis of the association between a recurrent pregnancy loss and the risk of developing pre-eclampsia in a subsequent pregnancy.


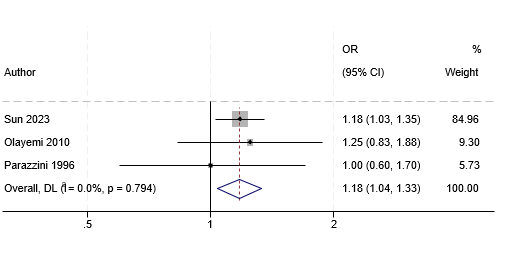


**OR: Odds ratio. CI: Confidence interval, p: P-value**

Supplementary Figure 7. Meta-analysis of the association between a spontaneous abortion and the risk of developing gestational hypertension in a subsequent pregnancy.


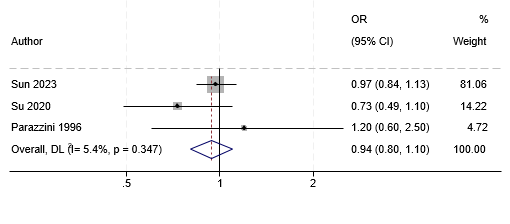


**OR: Odds ratio. CI: Confidence interval, p: P-value**

Supplementary Figure 8. Meta-analysis of the association between an induced abortion and the risk of developing gestational hypertension in a subsequent pregnancy.


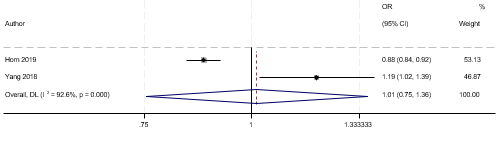


**OR: Odds ratio. CI: Confidence interval, p: P-value**

Supplementary Figure 9. Meta-analysis of the association between an induced abortion and the risk of developing non-pregnancy related hypertension.


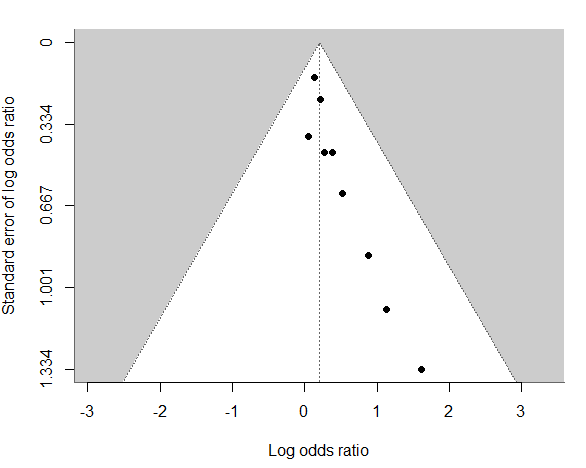


Supplementary Figure 10. Funnel plot with pseudo 95% confidence limits for the odds ratio of the association between a prior spontaneous abortion and the risk of developing gestational diabetes mellitus from the nine included studies.


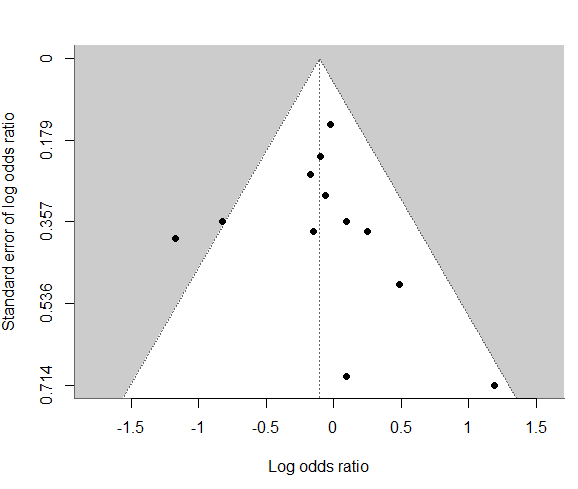


Supplementary Figure 11. Funnel plot with pseudo 95% confidence limits for the odds ratio of the association between a prior spontaneous abortion and the risk of developing pre-eclampsia from the 12 included studies.

References of the included studies (n=52)

1. Li TC, Yip BHK, Chen X. Recurrent miscarriage and risk of obstetric and perinatal complications in subsequent pregnancy: abridged secondary publication. *Hong Kong Medical Journal*. 2023;29(1):44-46.

2. Sun H, Mao J, Su X, Du Q. Impact of spontaneous abortion history and induced abortion history on perinatal outcomes of singleton pregnancies. *BMC Public Health*. 2023;23(1):2360-2360. doi:10.1186/s12889-023-17264-5

3. Vaajala M, Liukkonen R, Ponkilainen V, Kekki M, Mattila VM, Kuitunen I. Previous induced abortion or miscarriage is associated with increased odds for gestational diabetes: a nationwide register-based cohort study in Finland. Article. *Acta Diabetologica*. 2023;doi:10.1007/s00592-023-02047-6

4. Zhang J, Liu X, Rao L, et al. Adverse obstetric and perinatal outcomes of patients with history of recurrent miscarriage: a retrospective cohort study. *Fertility and Sterility*. 2023;120(3):626-634. doi:10.1016/j.fertnstert.2023.04.028

5. Askari M, Dadbinpour A, Ekraminasab S, Shukohifar M. Incidence and risk factors related to gestational diabetes mellitus among women in Yazd: A prospective cohort study. *World Journal of Peri & Neonatology* 2023;5(2):65-73.

6. Mohamedain A, Rayis DA, AlHabardi N, Adam I. Association between previous spontaneous abortion and preeclampsia: a case-control study. *BMC Pregnancy & Childbirth*. 2022;22(1):1-5. doi:10.1186/s12884-022-05053-8

7. Okoth K, Subramanian A, Chandan JS, et al. Long term miscarriage-related hypertension and diabetes mellitus. Evidence from a United Kingdom population-based cohort study. *PLoS One*. 2022;17(1):e0261769-e0261769. doi:10.1371/journal.pone.0261769

8. Taye H, Kabthymer RH, Hailu S, et al. Previous adverse pregnancy events as a predictor of gestational diabetes mellitus in Southern Ethiopia: a case control study. Article. *Current Medical Research and Opinion*. Jul 3 2022;38(7):1259-1266. doi:10.1080/03007995.2022.2083399

9. Zhao Y, Zhao Y, Fan K, Jin L. Association of history of spontaneous or induced abortion with subsequent risk of gestational diabetes. *JAMA Network Open*. 2022;5(3):e220944-e220944. doi:10.1001/jamanetworkopen.2022.0944

10. Ausbeck EB, Blanchard CT, Neely C, Tita AT, Szychowski JM, Harper LM. Perinatal outcomes in women with a history of recurrent pregnancy loss. *SMFM Fellowship Series Article*. 2021;

11. Huo Y, Cheng L, Wang C, et al. Associations between parity, pregnancy loss, and breastfeeding duration and risk of maternal type 2 diabetes: An observational cohort study. *Journal of Diabetes*. 2021;13(11):857-867. doi:10.1111/1753-0407.13176

12. Roepke ER, Christiansen OB, Källén K, Hansson SR. Women with a history of recurrent pregnancy loss are a high-risk population for adverse obstetrical outcome: A retrospective cohort study. *Journal of Clinical Medicine*. 2021;10(2):1-12. doi:10.3390/jcm10020179

13. Stitterich N, Shepherd J, Koroma MM, Theuring S. Risk factors for preeclampsia and eclampsia at a main referral maternity hospital in Freetown, Sierra Leone: a case-control study. *BMC Pregnancy and Childbirth*. 2021;21(1):1-413. doi:10.1186/s12884-021-03874-7

14. Ali N, Elbarazi I, Ghazal-Aswad S, et al. Impact of recurrent miscarriage on maternal outcomes in subsequent pregnancy: The mutaba’ah study. *International Journal of Womens Health*. 2020;12:1171-1179. doi:10.2147/IJWH.S264229

15. Christians JK, Huicochea Munoz MF. Pregnancy complications recur independently of maternal vascular malperfusion lesions. *PloS One*. 2020;15(2):e0228664-e0228664. doi:10.1371/journal.pone.0228664

16. Egerup P, Mikkelsen AP, Kolte AM, et al. Pregnancy loss is associated with type 2 diabetes: a nationwide case–control study. *Diabetologia*. 2020;63(8):1521-1529. doi:10.1007/s00125-020-05154-z

17. Peters SAE, Yang L, Guo Y, et al. Pregnancy, pregnancy loss and the risk of diabetes in Chinese women: findings from the China Kadoorie Biobank. *European Journal of Epidemiology*. 2019;35(3):295-303. doi:10.1007/s10654-019-00582-7

18. Sembiring RL, Mappaware NA, Usman AN. Relationship between characteristics and obstetric history with hypertension in pregnancy. *Enfermería Clínica*. 2019;30(S2):31-34. doi:https://doi.org/10.1016/j.enfcli.2019.07.022

19. Su Y, Xie X, Zhou Y, et al. Association of induced abortion with hypertensive disorders of pregnancy risk among nulliparous women in China: a prospective cohort study. *Scientific Reports*. 2020;10(1):5128-5128. doi:10.1038/s41598-020-61827-0

20. Ticconi C, Pietropolli A, Specchia M, et al. Pregnancy-Related Complications in Women with Recurrent Pregnancy Loss: A Prospective Cohort Study. *Journal of Clinical Medicine*. 2020;9(9):2833. doi:10.3390/jcm9092833

21. Unnikrishnan B, Rathi P, Bhat SK, et al. Risk factors of gestational diabetes mellitus: A hospital-based pair-matched case-control study in coastal south india. *South African Journal of Obstetrics and Gynaecology*. 2020;26(1):13-17. doi:10.7196/SAJOG.2020.v26i1.1518

22. Horn J, Tanz LJ, Stuart JJ, et al. Early or late pregnancy loss and development of clinical cardiovascular disease risk factors: a prospective cohort study. *BJOG: An International Journal of Obstetrics & Gynaecology*. 2019;126(1):33-42. doi:10.1111/1471-0528.15452

23. Cozzolino M, Rizzello F, Riviello C, Romanelli C, Coccia Elisabetta M. Ongoing pregnancies in patients with unexplained recurrent pregnancy loss: adverse obstetric outcomes. *Human Fertility* 2019;22(3):219-225. doi:10.1080/14647273.2018.1475754

24. Feleke BE. Determinants of gestational diabetes mellitus: a case-control study. *Journal of Maternal-Fetal and Neonatal Medicine*. 2018;31(19):2584-2589. doi:10.1080/14767058.2017.1347923

25. Lao TT, Hui ASY, Law L-W, Sahota DS. Prior abortion history and pregnancy hypertensive disorders in primiparous gravidae. *Pregnancy Hypertension*. 2018;14:168-173. doi:10.1016/j.preghy.2018.10.001

26. Liu B, Song L, Li H, et al. History of spontaneous miscarriage and the risk of diabetes mellitus among middle-aged and older Chinese women. *Acta Diabetologica*. 2018;55(6):579-584. doi:10.1007/s00592-018-1125-z

27. Yang Q, Song C, Jiang J, et al. Association of reproductive history with hypertension and prehypertension in Chinese postmenopausal women: A population-based cross-sectional study. *Hypertension Research*. 2018;41(1):66-74. doi:10.1038/hr.2017.86

28. Liu L-Y, Zhang Y-L, Li L. Risk factor of gestational diabetes among healthy Chinese women: an observational study. *Biomedical Research*. 2017;28(5):2126-2130.

29. Sepidarkish M, Almasi‐Hashiani A, Maroufizadeh S, Vesali S, Pirjani R, Samani RO. Association between previous spontaneous abortion and pre‐eclampsia during a subsequent pregnancy. *International Journal of Gynecology & Obstetrics*. 2017;136(1):83-86. doi:10.1002/ijgo.12008

30. Su Y-y, Jin-zhi Zhang, Wang F. Risk factors and adverse outcomes of preeclampsia: a tertiary care centrebased study in China. *Biomedical Research* 2017;28(3):1262-1265.

31. AlKasseh ASM, Zaki NM, Aljeesh YI, Soon LK. Risk factors of gestational diabetes mellitus in the refugee population in Gaza Strip: a case-control study. *Eastern Mediterranean Health Journal*. 2014;19 Suppl 3:S12-S18.

32. Gunnarsdottir JMD, Stephansson OMDP, Cnattingius SMDP, Åkerud HMDP, Wikström A-KMDP. Risk of placental dysfunction disorders after prior miscarriages: a population-based study. *American Journal of Obstetrics and Gynecology*. 2014;211(1):34.e1-34.e8. doi:10.1016/j.ajog.2014.01.041

33. Makhlouf MA, Clifton RG, Roberts JM, et al. Adverse pregnancy outcomes among women with prior spontaneous or induced abortions. *American Journal of Perinatology*. 2014;31(9):765-772. doi:10.1055/s-0033-1358771.

34. Parker SE, Gissler M, Ananth CV, Werler MM. Induced abortions and the risk of preeclampsia among nulliparous women. *American Journal of Epidemiology*. 2014;182(8):663-669. doi:10.1093/aje/kwv184

35. Ranthe MF, Andersen EAW, Wohlfahrt J, Bundgaard H, Melbye M, Boyd HA. Pregnancy loss and later risk of Atherosclerotic disease. *Circulation*. 2013;127(17):1775-1782. doi:10.1161/CIRCULATIONAHA.112.000285

36. Kharazmi E, Lukanova A, Teucher B, Groß M-L, Kaaks R. Does pregnancy or pregnancy loss increase later maternal risk of diabetes? *European Journal of Epidemiology*. 2012;27(5):357-366. doi:10.1007/s10654-012-9683-9

37. Weintraub AYMD, Sergienko R, Harlev AMD, et al. An initial miscarriage is associated with adverse pregnancy outcomes in the following pregnancy. *American Journal of Obstetrics and Gynecology*. 2011;205(3):286.e1-286.e5. doi:10.1016/j.ajog.2011.06.053

38. Bhat M, Ramesha KN, Sarma SP, Menon S, Sowmini CV, Ganesh Kumar S. Determinants of gestational diabetes mellitus: A case control study in a district tertiary care hospital in south India. *International Journal of Diabetes in Developing Countries*. 2010;30(2):91-96. doi:10.4103/0973-3930.62599

39. Olayemi O, Strobino D, Adedapo K, Aimakhu C, Odukogbe A-t, Salako B. Influence of previous abortions and new paternity on the risk of hypertension in nulliparous parturients in Ibadan: A cohort study: Abortion and hypertension in pregnancy in Nigeria. *The Journal of Obstetrics and Gynaecology Research*. 2010;36(5):965-969. doi:10.1111/j.1447-0756.2010.01268.x

40. Trogstad L, Magnus P, Skjærven R, Stoltenberg C. Previous abortions and risk of pre-eclampsia. *International Journal of Epidemiology*. 2008;37(6):1333-1340. doi:10.1093/ije/dyn167

41. Trogstad L, Magnus P, Moffett A, Stoltenberg C. The effect of recurrent miscarriage and infertility on the risk of pre-eclampsia. *BJOG: An International Journal of Obstetrics & Gynaecology*. 2009;116(1):108-113. doi:10.1111/j.1471-0528.2008.01978.x

42. Yang H, Wei Y, Gao X, et al. Risk factors for gestational diabetes mellitus in Chinese women-a prospective study of 16286 pregnant women in China. *Diabetic Medicine*. 2009;26(11):1099-1104. doi:10.1111/j.1464-5491.2009.02845.x

43. Bhattacharya S, Townend J, Shetty A, Campbell D, Bhattacharya S. Does miscarriage in an initial pregnancy lead to adverse obstetric and perinatal outcomes in the next continuing pregnancy? *BJOG: An International Journal of Obstetrics & Gynaecology*. 2008;115(13):1623-1629. doi:10.1111/j.1471-0528.2008.01943.x

44. Sheiner E, Levy A, Katz M, Mazor M. Pregnancy outcome following recurrent spontaneous abortions. *European Journal of Obstetrics & Gynecology and Reproductive Biology*. 2005;118(1):61-65. doi:10.1016/j.ejogrb.2004.06.015

45. Vanek M, Sheiner E, Levy A, Mazor M. Chronic hypertension and the risk for adverse pregnancy outcome after superimposed pre-eclampsia. *International Journal of Gynecology and Obstetrics*. 2004;86(1):7-11. doi:10.1016/j.ijgo.2004.03.006

46. Saftlas AF, Levine RJ, Klebanoff MA, et al. Abortion, changed paternity, and risk of preeclampsia in nulliparous women. *American Journal of Epidemiology*. 2003;157(12):1108-1114. doi:10.1093/aje/kwg101

47. Xiong X, Fraser WD, Demianczuk NN. History of abortion, preterm, term birth, and risk of preeclampsia: A population-based study. *American Journal of Obstetrics and Gynecology*. 2002;187(4):1013-1018. doi:10.1067/mob.2002.126282

48. Eras JL, Saftlas AF, Triche E, Hsu C-D, Risch HA, Bracken MB. Abortion and Its Effect on Risk of Preeclampsia and Transient Hypertension. *Epidemiology*. 2000;11(1):36-43. doi:10.1097/00001648-200001000-00009

49. Sibai BM, Ewell M, Levine RJ, et al. Risk factors associated with preeclampsia in healthy nulliparous women. *American Journal of Obstetrics and Gynecology*. 1997;177(5):1003-1010. doi:10.1016/S0002-9378(97)70004-8

50. Parazzini F, Bortolus R, Chatenoud L, et al. Risk factors for pregnancy-induced hypertension in women at high risk for the condition. *Epidemiology* 1996;7(3):306-308. doi:10.1097/00001648-199605000-00016

51. Abi-Said D, Annegers JF, Combs-Cantrell D, Frankowski RF, Willmore LJ. Case-control study of the risk factors for eclampsia. *American Journal of Epidemiology*. 1995;142(4):437-441. doi:10.1093/oxfordjournals.aje.a117652

52. Eskenazi B, Fenster L, Sidney S. A Multivariate Analysis of Risk Factors for Preeclampsia. *JAMA*. 1991;266(2):237-241. doi:10.1001/jama.1991.03470020063033
